# Minimizing Mitogenic Potency of Insulin Analogues Through Modification of a Disulfide Bond

**DOI:** 10.3389/fendo.2022.907864

**Published:** 2022-06-27

**Authors:** Shee Chee Ong, Alessia Belgi, Allanah L. Merriman, Carlie A. Delaine, Bianca van Lierop, Sofianos Andrikopoulos, Andrea J. Robinson, Briony E. Forbes

**Affiliations:** ^1^ Discipline of Medical Biochemistry, Flinders Health and Medical Research Institute, Flinders University of South Australia, Bedford Park, SA, Australia; ^2^ School of Chemistry, Monash University, Clayton, VIC, Australia; ^3^ Department of Medicine, University of Melbourne, Parkville, VIC, Australia

**Keywords:** insulin, dicarba insulin, insulin receptor, biased signalling agonists, mitogenic, extracellular-signal-regulated kinase (ERK), glucose metabolism, cell signalling

## Abstract

The mechanisms by which insulin activates the insulin receptor to promote metabolic processes and cellular growth are still not clear. Significant advances have been gained from recent structural studies in understanding how insulin binds to its receptor. However, the way in which specific interactions lead to either metabolic or mitogenic signalling remains unknown. Currently there are only a few examples of insulin receptor agonists that have biased signalling properties. Here we use novel insulin analogues that differ only in the chemical composition at the A6–A11 bond, as it has been changed to a rigid, non-reducible C=C linkage (dicarba bond), to reveal mechanisms underlying signaling bias. We show that introduction of an A6-A11 *cis-*dicarba bond into either native insulin or the basal/long acting insulin glargine results in biased signalling analogues with low mitogenic potency. This can be attributed to reduced insulin receptor activation that prevents effective receptor internalization and mitogenic signalling. Insight gained into the receptor interactions affected by insertion of an A6-A11 *cis-*dicarba bond will ultimately assist in the development of new insulin analogues for the treatment of diabetes that confer low mitogenic activity and therefore pose minimal risk of promoting cancer with long term use.

## Introduction

Insulin acts *via* the insulin receptor (IR) that belongs to the receptor tyrosine kinase family. Signalling *via* the IR promotes essential metabolic processes required for maintaining glucose homeostasis. People with Type 1 diabetes, and many with late-stage Type 2 diabetes, use rapid acting insulin analogues to control circulating glucose levels in the postprandial phase (e.g. insulin lispro or KP insulin; Humalog^®^, Eli Lilly & Co.) and the basal phase (e.g. insulin glargine; Lantus^®^, Sanofi). While these analogues are generally effective there is a need for new analogues with improved properties, including rapid acting analogues with faster onset to minimise postprandial glucose excursions and long acting analogues with limited mitogenic properties.

Since the termination of preclinical safety testing of the postulated highly mitogenic insulin X10 (1) (HisB10Asp insulin), emphasis has been placed on reducing any potential carcinogenic risk of newly developed insulin mimetics (2). While there are no definitive reports to suggest insulin analogues currently in clinical use promote higher cancer risk, controversial discussions surrounding the complex relationships between insulin therapy, diabetes and cancer continue (2–7). Ideally, future insulin analogues should be selected to have optimal signalling traits – metabolically effective and not mitogenically active.

**Figure 1:**
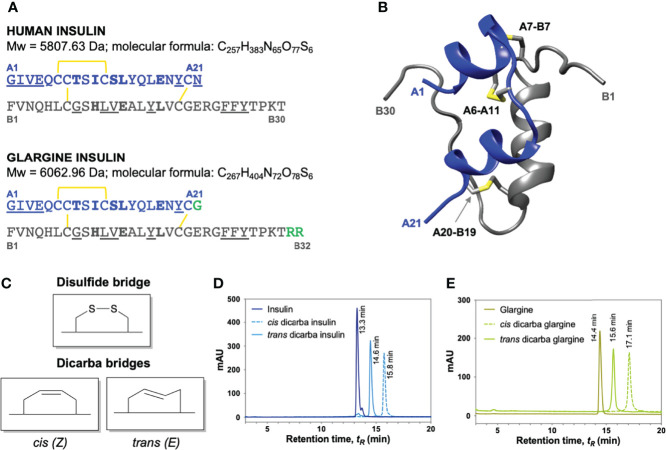
Insulin, insulin glargine and dicarba insulin analogues. **(A)** Primary sequence comparison of human insulin (top) and insulin glargine (*bottom*). Both consist of A (*blue*) and B (*grey*) chains stabilized by three disulfide bridges (yellow). *Underlined* are site 1-binding residues and *bold* are site 2-binding residues (8). Long-acting insulin glargine has a substitution of AsnA21 ➛ GlyA21 residue and an addition of ArgB31 ArgB32 residues on the *C*-terminal end of B chain (highlighted in *green*). **(B)** Ribbon diagram of human insulin (2-Zn-coordinated T_6_ conformation PDB entry 1MSO) showing the location of the three α-helices (*A chain: blue; B chain: grey*) and the three disulfide bonds (*yellow*). **(C)** Schematic diagram of native cysteine and isomeric *cis-* and *trans-*dicarba bridges. RP-HPLC chromatograms of **(D)** insulin, *cis-*dicarba insulin and *trans-*dicarba insulin (9); **(E)** insulin glargine, *cis-*dicarba dicarba glargine and *trans-*dicarba glargine. Analysis of the final synthesis products by mass spectrometry confirmed the correct masses of dicarba insulin (5766 Da) and dicarba insulin glargine (6021 Da).

In order to design such an insulin analogue an understanding of the mechanisms of IR binding and activation that regulates signalling selectivity is required. Insulin is a two-chain polypeptide comprising a 21-residue A chain and a 30-residue B chain. The secondary structure of insulin consists of three α-helices, two within the A chain (A1 to A8 and A12 to A18) and a single α-helix within the central segment of the B chain (B9 to B19). The three-dimensional structure of insulin is facilitated by two intra-chain (CysA7-CysB7 and CysA20-CysB19) and one inter-chain (CysA6-CysA11) disulfide bonds. High affinity interaction of insulin with the IR involves determinants arising from insulin’s overall structure and from specific side chains located across two distinct surfaces of the insulin molecule (high affinity site 1 and low affinity site 2) (**Figures 1A, B**). These interact with two distinct regions on the receptor that have been characterized using site-directed mutagenesis and structural studies (8, 10–17), with IR site 1 comprising the L1 domain of one IR monomer and the αCT peptide of the second monomer (αCT’) making contact with insulin site 1 residues [reviewed in (18)]. The significance of the IR site 2 on the Fn-III-1’ is still not entirely clear but it may represent the first site of insulin contact (18). Interaction of insulin with the IR leads to substantial IR conformational change promoting IR tyrosine kinase activation, IR autophosphorylation (including juxtamembrane domain Tyr960; tyrosine kinase activation loop Tyr1148, Tyr1150, Tyr1151; C-terminal domain Tyr1316 and Tyr1322), recruitment of adapter molecules (including insulin receptor substrates, IRS-1 and IRS-2, and Shc) and subsequent downstream signalling (19).

Our limited understanding of the specific mechanisms that mediate insulin’s metabolic and mitogenic responses has been derived from characterization of a small number of biased ligands. For example, although the therapeutic promise of insulin X10 was short-lived, it is an important mitogenically-biased insulin agonist, the study of which has revealed several key insights that may explain insulin’s mitogenicity. Not only does insulin X10 have an increased binding affinity for the growth-promoting insulin-like growth factor receptor 1 (IGF-1R) but it also has a reduced rate of dissociation from the IR compared to insulin (20, 21). Its prolonged receptor residence time correlates with a sustained phosphorylation of the IR, an increased rate of receptor internalization, and enhanced mitogenic signalling (20). The basal analogue insulin glargine (GlyA21, ArgB30, ArgB31 insulin) was initially suspected to be a mitogenically biased insulin. It also binds to the IGF-1R with higher affinity and promotes an increased cell proliferation response in comparison to native insulin (22). Interestingly, however, the insulin receptor isoform A (IR-A) glargine’s dissociation kinetics do not differ from native insulin (21) and it is equipotent in promoting mitogenic cell signalling (20, 23). Conversely, the metabolically-biased S597 insulin mimetic peptide, extensively studied by Jensen et al. (24) and recently in our laboratory (25), fails to promote IR internalization. This correlates strongly with its reduced mitogenic potency compared to full metabolic activity (24, 25). Collectively, this evidence suggests IR signalling bias is influenced by ligand IR dissociation kinetics and IR internalization rates (25, 26).

Until now the studies investigating IR signalling bias have used insulin analogues with specific amino acid changes (like insulin X10), agonistic monoclonal antibodies (27), aptamers (28) or insulin mimetic peptides (24, 25). Although insulin mimetics have been useful to study mechanisms of signalling bias some, including the single chain S597 peptide, activate the IR *via* binding to sites distinct from the previously defined IR binding sites 1 and 2 (28, 29). In this study, we compare insulin with an analogue in which the A6–A11 disulfide bond has been replaced with an unsaturated dicarba bond (*cis-*dicarba insulin). Such an analogue provides a unique and important opportunity to compare the differential signalling properties between a peptide that has an identical amino acid sequence but differs only in the chemical composition at the A6–A11 bond. The installed dicarba bond is a rigid, non-reducible C=C linkage (see **Figure 1C**). Previously, we reported that the *cis* isomer of dicarba insulin binds the IR with equal affinity to insulin, promotes equivalent metabolic activity but is poor at activating a mitogenic response (9, 30). With this in mind, we first sought to determine whether the mitogenic activity of insulin glargine could be reduced through introduction of an equivalent dicarba bond. Here we show that *cis-*dicarba glargine does in fact act as a biased agonist with poor mitogenic potential. Subsequently, to understand the mechanism underlying the biased action of *cis-*dicarba insulin and *cis-*dicarba glargine, we investigate the relationship between phosphorylation kinetics of the IR, key IR adaptor proteins and downstream signalling molecules with IR internalization and subsequent biological activities. Our findings provide key insight into mechanisms underlying the activation of metabolic and mitogenic signalling *via* the IR using a structural mimetic of the insulin molecule in which the sequence is unchanged but for the installation of a conformationally constrained A6-A11 bridge.

## Results

### Chemical Synthesis of Cis-Dicarba Glargine Insulin

As our previous studies identified that *cis-*[Δ^4^A6,11]-dicarba human insulin (*cis-*dicarba insulin, with a non-reducible C=C linkage) is a biased IR agonist with reduced mitogenic potential (9, 30). We therefore sought to investigate whether the increased mitogenic potential of insulin glargine could be reduced through introduction of an analogous A6–A11 dicarba bond.

The *cis-* and *trans-*configured dicarba glargine insulin A chains, in which the A6–A11 intra-chain S-S bond is replaced by a C=C dicarba bond (**Figure 1C**), were synthesized as previously described using an RCM and SPPS-catalysis approach (9, 31). The modified dicarba glargine insulin A chains were then combined with the requisite insulin B chain to provide *cis-* and *trans-*isomers of c[Δ^4^A6,11]-dicarba glargine insulin. The synthesis of dicarba glargine is summarized in **Figure S1**. Isomers were separated at the final RP-HPLC step (**Figures 1D, E**) and correct synthesis of products was confirmed by mass spectrometry (*cis-*dicarba glargine insulin, *m/z* 6020.96).

### Receptor Binding & Activation Analyses

**Figure 2:**
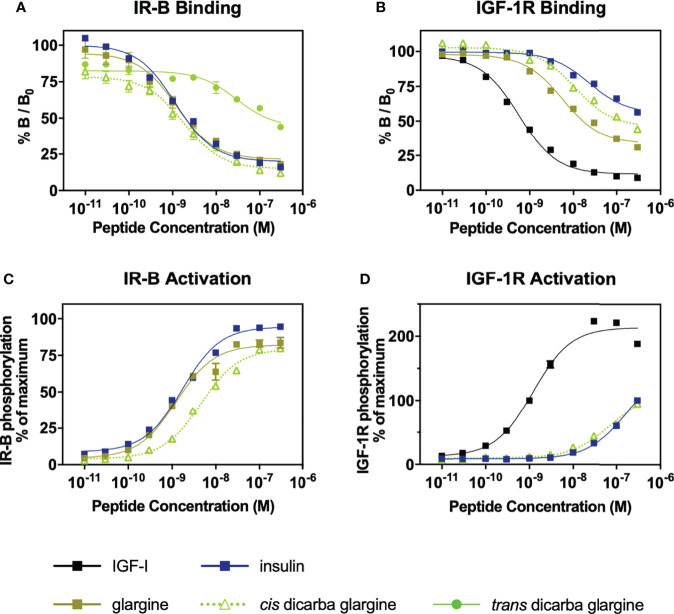
Receptor binding and activation of insulin, glargine and dicarba glargine analogues. **(A)** Competition binding of insulin, glargine and dicarba glargine analogues with europium-labelled insulin for the IR-B and **(B)** with europium-labelled IGF-I for the IGF-1R. Results are expressed as a percentage of binding in the absence of competing ligand (%B/B_0_). **(C)** Activation of IR-B and **(D)** IGF-1R by increasing concentrations of each insulin analogue (10 min stimulation) is expressed as a percentage of the maximal receptor phosphorylation induced by insulin. All data are the mean ± S.E.M. n = at least 3 independent experiments. Error bars are shown when greater than the size of the symbols. Competition binding of insulin, glargine and dicarba glargine analogues with europium-labelled insulin for the IR-A were also performed (see **Figure S2**).

The binding affinities of the dicarba glargine insulin analogues for the IR isoform B (IR-B), IGF-1R and IR isoform A (IR-A) were determined using competition binding assays (**Figures 2A, B**; **Figure S2**; **Table 1**). Similar to the effect of dicarba bonds on native insulin (9) and KP insulin (30), the *cis*-isomer of dicarba glargine was able to effectively bind to the IR while the *trans* isomer bound poorly. The *trans-*dicarba glargine was subsequently excluded from further receptor binding or activity assays due to its poor IR-B binding affinity. Insulin glargine bound to the IRs with a potency similar to or slightly lower than native insulin (88% *via* IR-B, ~35% *via* IR-A, compared to insulin), consistent with the literature [40-86% IR-A, 50-85% IR-B (21–23, 32)]. Additionally, glargine showed increased affinity for the IGF-1R in comparison with insulin [480% of native insulin reported in this study; 460–650% in (21, 23)]. Compared to insulin, *cis-*dicarba glargine showed a similar affinity for IR-B and reduced affinity for IR-A (~2.5 fold lower) (**Figure 2A**; **Figure S2**; **Table 1**). Whilst *cis-*dicarba glargine had a higher affinity than insulin for the IGF-1R (~260% of insulin) its affinity compared to glargine was significantly lower (~2 fold).

**Table 1:** Binding affinities of IR-B and IGF-1R.

	IR-B		IGF-1R	
	IC_50_ (nM)	Affinity (% Insulin ± SEM)	IC_50_ (nM)	Affinity (% Insulin ± SEM)
Insulin	1.07 ± 0.09	100	> 300	100
*cis* dicarba insulin^b^	1.07 ± 0.30	100 ± 26^ns^	> 300	~100
*trans* dicarba insulin	40.4 ± 7.2^a^	2.7 ± 0.3****	–	–
Glargine	1.22 ± 0.13	88 ± 9 ^ns^	6.71 ± 3.9	> 484 ± 281 ****
*cis* dicarba glargine	1.95 ± 0.38	55 ± 10****	11.5 ± 3.16	> 261 ± 76 ****
*trans* dicarba glargine	30.8 ± 15	3.48 ± 3.6****	–	–
IGF-I	–	–	0.60 ± 0.07	4704 ± 468 ****

n = 3 or more.

a n=2.

^b^= dicarba insulin data from (9).

- not performed.

^ns^non-statistically significant.

****P ≤ 0.0001 (2-way ANOVA; Dunnett’s multiple comparison). Errors shown are S.E.M.

Competition for europium-labelled insulin and IGF-I binding to the IR-B and IGF-1R, respectively, by insulin, insulin glargine, dicarba insulin analogues and IGF-I. These data were derived from IR-B receptor binding curves in **Figure 2A** and IGF-1R receptor binding curves in **Figure 2B**.

Using the kinase insulin receptor activation assay (KIRA) that detects phosphorylated tyrosine on immunocaptured, activated receptors (33), glargine insulin activated the IR-B with a potency similar to native insulin (**Figure 2C**) in line with previous reports (32). Of interest, the *cis-*dicarba glargine was shown to activate the IR-B with a ~3.5-fold reduced potency compared to glargine (**Figure 2C**) despite its similar affinity for the IR-B. Both insulin and *cis-*dicarba glargine were equipotent but poorly activated the IGF-1R (**Figure 2D**). This contrasts with *cis-*dicarba insulin which was equipotent to insulin in both binding and activation of IR-B and IGF-1R (9).

### *In Vitro* Metabolic and Mitogenic Activity

**Figure 3:**
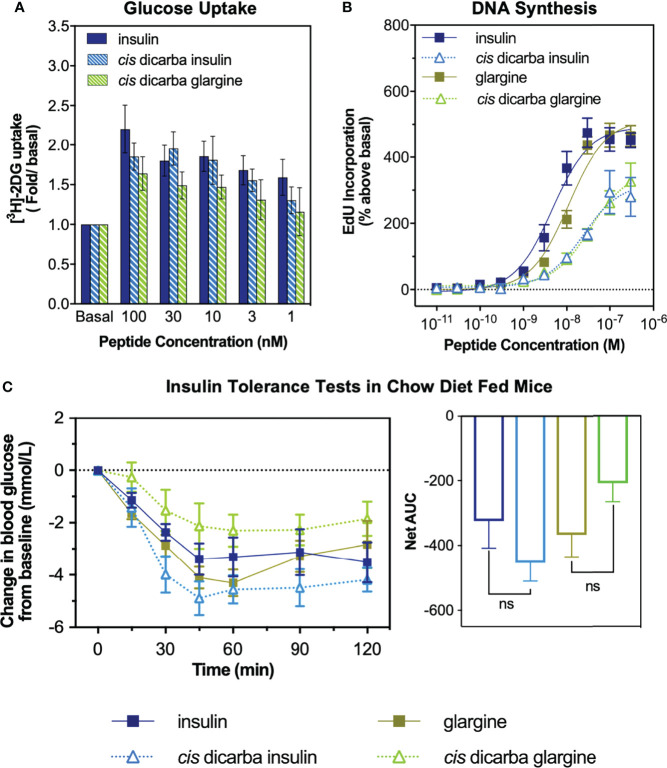
*In vitro* metabolic and mitogenic studies of insulin, glargine and *cis*-dicarba analogues. **(A)** Glucose uptake stimulated by increasing concentrations of insulin, *cis-*dicarba insulin or *cis-*dicarba glargine is expressed as fold 2-deoxyglucose (2-DG) uptake (pmol/min/mg) above basal. Insulin *vs cis*-dicarba insulin *vs cis-*dicarba glargine (ns) (paired *T-test*). **(B)** DNA synthesis in response to increasing concentrations of stimulating insulin analogue is shown as percentage incorporation of 5-Ethynyl-2’-uridine (EdU) above basal. All data in **(A)** and **(B)** are the mean ± S.E.M. n = at least 3 independent experiments. **(C)** Insulin tolerance tests were conducted in mice fed on a normal diet (chow). Insulin, glargine, *cis*-dicarba insulin or *cis-*dicarba glargine (0.75 IU/kg) were administered through intraperitoneal injection (ip) under non-fasting conditions and tail vein blood glucose was measured *via* glucose meter at indicated times. n = 5-6 per group. Blood glucose levels are expressed as change over basal levels (mmol/L). AUC, area under the curves. Chow diet: insulin *vs cis-*dicarba insulin^ns^; glargine *vs cis-*dicarba glargine^ns^ (paired *T-test*). Error bars for all graphs are shown when greater than the size of the symbols.

The *cis-*dicarba glargine was equally potent to *cis-*dicarba insulin and native insulin in promoting glucose uptake by NIH3T3-L1 adipocytes, despite showing a statistically insignificant trend towards lower potency that corresponds to its slightly lower IR-B binding and activation potencies (**Figure 3A**). This suggests that introduction of the dicarba linkage has little effect on the ability to activate metabolic signalling pathways upon IR-B binding.

As a measure of *in vitro* mitogenic activity, DNA synthesis was observed in L6 rat skeletal myoblasts overexpressing human IR-A (L6 IR-A cells). Glargine was equipotent (within ~2-fold) to native insulin in promoting DNA synthesis (**Figure 3B**), consistent with previous research (32). The introduction of the dicarba bond to insulin, reduced mitogenic potency by ~5-fold, in line with our previous report (9). Importantly, the *cis-*dicarba glargine analogue demonstrated a marked reduction in potency in promoting DNA synthesis when compared to native insulin and glargine (~8-fold and ~3-fold respectively) (**Figure 3B**). Notably, there was a discrepancy between the abilities of both *cis-*dicarba insulin and *cis-*dicarba glargine to activate DNA synthesis *via* the IR-A and their IR-A binding affinities [**Figure 3B**; **Figure S2** (9)]. The introduction of the A6-A11 dicarba bond significantly reduced mitogenic activity by ~8-10 fold, while only binding to the IR-A with ~2-fold lower affinity compared to native insulin. This suggests that introduction of the dicarba bond to insulin or glargine is having a similar effect on the ability of both insulins to bind and activate the IR. In summary, *cis-*dicarba glargine retains a metabolic potency similar to insulin that allows the analogue to effectively promote glucose uptake *via* stimulation of IR-B but has a reduced mitogenic potency *via* the IR-A (**Figure 3**).

### Insulin Tolerance Test

All insulin analogues lowered blood glucose to a similar extent (**Figure 3C**). Similar to the effect of A6–A11 *cis-*dicarba bridge in native insulin (9) and KP insulin, the introduction of the A6–A11 dicarba bond into glargine resulted in an analogue that effectively lowered blood glucose levels in chow fed mice (**Figure 3C**). As was seen with *cis*-dicarba insulin, there was no statistical difference in the abilities of insulin glargine and *cis-*dicarba glargine to lower blood glucose over the 2 hours post-treatment, as was seen with *cis-*dicarba insulin compared with insulin.

### Dose-Response Effects on P13K and MAPK Signalling

**Figure 4:**
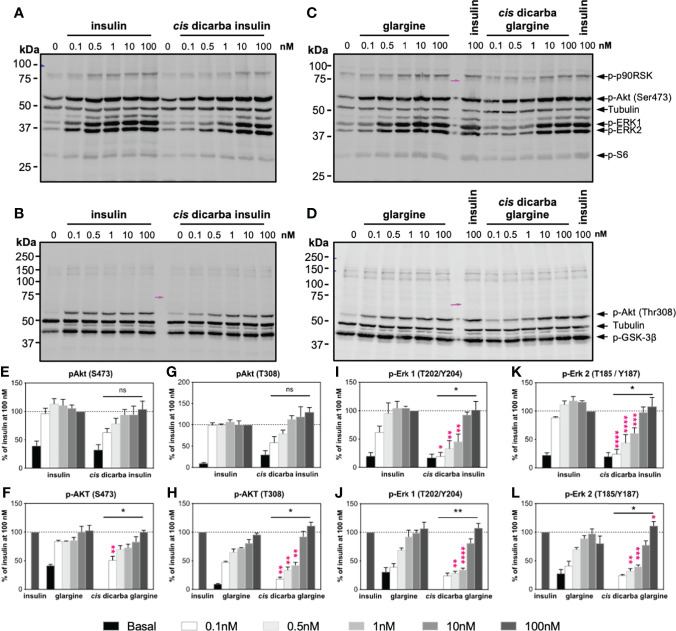
*Cis-*dicarba insulin and *cis-*dicarba glargine insulin exhibit dose-dependent signaling bias *via* IR-A overexpressing cells. Serum starved IR-A overexpressing L6 myoblasts (hIR-A L6) were stimulated for 10 min with increasing concentrations (0, 0.1, 0.5 1, 10 and 100 nM) of **(A–B)** insulin or *cis-*dicarba insulin, **(C, D)** insulin glargine or *cis-*dicarba glargine. Pink arrow aligns to 75 kDa marker (representative blots of n = at least 3 independent experiments). **(E-L)** Quantitation of western blots. Phosphorylation levels are expressed as percentage of level detected when cells were stimulated with 100 nM of insulin for 10 min; normalized to β-tubulin: **(E, F)** Akt (phospho-Ser473), **(G, H)** Akt (phospho-Thr308), **(I, J)** ERK 1 (phospho-Thr202/Tyr204) and **(K, L)** ERK 2 (phospho-Thr185/Tyr187). Quantitation of other phosphorylated proteins are included in **Figure S3**. All data are the mean ± *S.E.M*. Statistical significance of the overall difference in phosphorylation levels stimulated by *cis-*dicarba insulin compared to native insulin or *cis-*dicarba glargine compared to glargine insulin were determined *via 2*-way ANOVA (bars above graph); difference comparing each stimulating concentration (pink asterisks) were further analyzed using Holm-Sidak test. ns: non-significant; * (P ≤ 0.05), ** (P ≤ 0.01); *** (P ≤ 0.001); **** (P ≤ 0.0001). Dose-dependent signalling analyses were also performed in IR-B overexpressing cells (see **Figures S4–S5**).

To investigate the basis of *cis-*dicarba insulin and *cis-*dicarba glargine signalling bias towards metabolic activity, activation of the molecules downstream of the IR involved in metabolic (PI3K/Akt) and mitogenic (MAPK) signalling was measured. Dose-response effects of insulin, glargine and the respective *cis-*dicarba analogues on these pathways were investigated using both L6 IR-A cells (**Figure 4** and **Figure S3**) and human IR-B overexpressing, IGF-1R-negative fibroblasts (R^-^IR-B cells) (**Figures S4** and **S5**).

Overall, in dose-response activation experiments, the *cis-*dicarba insulin and *cis-*dicarba glargine were significantly less effective than insulin or glargine, respectively, in activating the MAPK pathway. Such differential potencies were seen in both tested cell types but were more evident in the L6 IR-A cells (**Figure 4** and **Figure S3**) than in IR-B overexpressing fibroblasts (**Figures S4** and **S5**). A mitogenic response *via* the MAPK pathway is regulated by phosphorylation of ERK1/2 and p90RSK. Thus, activation and phosphorylation of ERK 1 (Thr202/Tyr204), ERK 2 (Thr185/Tyr187) and its downstream p90RSK (Ser380) were measured. Activation of IR-A receptors with *cis-*dicarba insulin (**Figures 4A, I, K** and **Figure S3C**) and *cis-*dicarba glargine (**Figures 4C, J, L** and **S3D**) resulted in significantly reduced phosphorylation levels of ERK 1, ERK 2 and p90RSK between concentration ranges of 0.1–10 nM at t = 10 min (p* ≤ 0.05 to p**** ≤ 0.0001). Similarly, activation of IR-B with both *cis-*dicarba insulins also showed dose-dependent lower phosphorylation levels of ERK 1 and ERK 2 (**Figures S4A–D** and **I–L**).

Compared to the MAPK pathway, the difference in activation of the PI3K/Akt pathway between native parent peptides and their respective *cis-*dicarba analogues was less evident. Therefore, the phosphorylation of Akt (p-Akt Thr308 and p-Akt Ser473) and S6 ribosomal protein (Ser235/236) was measured as a readout of the PI3K/Akt pathway. There was a reduced ability of *cis-*dicarba glargine but not *cis-*dicarba insulin to stimulate phosphorylation of Akt at Thr308 on L6 IR-A (**Figures 4G, H**; (p ≤ 0.01)) and R^-^IR-B cells (**Figures S4G, H**). The levels of Akt phosphorylation at Ser473 upon *cis-*dicarba insulin (IR-A: **Figures 4A, E**; IR-B: **Figures S4A, E**) or dicarba glargine (IR-A: **Figures 4C, F**; IR-B: **Figures S4C, F**) stimulation were generally similar to their parent peptides; with significant difference only apparent at the lowest concentration (0.1 nM; p* ≤ 0.05 to p** ≤ 0.01).

Downstream of Akt, phosphorylation of GSK-3β at Ser9 inhibits its activity and in turn promotes glycogen synthesis (34). Maximal phosphorylation of GSK-3β Ser9 was achieved even at the lowest concentrations used (0.1 nM) for all analogues, meaning that no potency differences between analogues could be detected for GSK-3β activation (IR-A: **Figures 4B, D**, and **Figures S3A, B**; IR-B: **Figures S4B, D** and **S5A, D**). In addition, in R^-^IR-B cells there was no difference in the abilities of all peptides to stimulate phosphorylation of AS160, an important effector of the PI3K/Akt pathway that promotes glucose uptake (**Figures S5G, H**). However, phosphorylation of AS160 was not able to be detected in L6 IR-A cells within this study.

In summary, *cis-*dicarba insulin and *cis-*dicarba glargine were significantly less potent at activating the MAPK pathway in a dose-dependent manner compared to insulin while maintaining equal potency in activating the PI3K/Akt pathway.

### Kinetics of PI3K/Akt and MAPK Signalling

**Figure 5:**
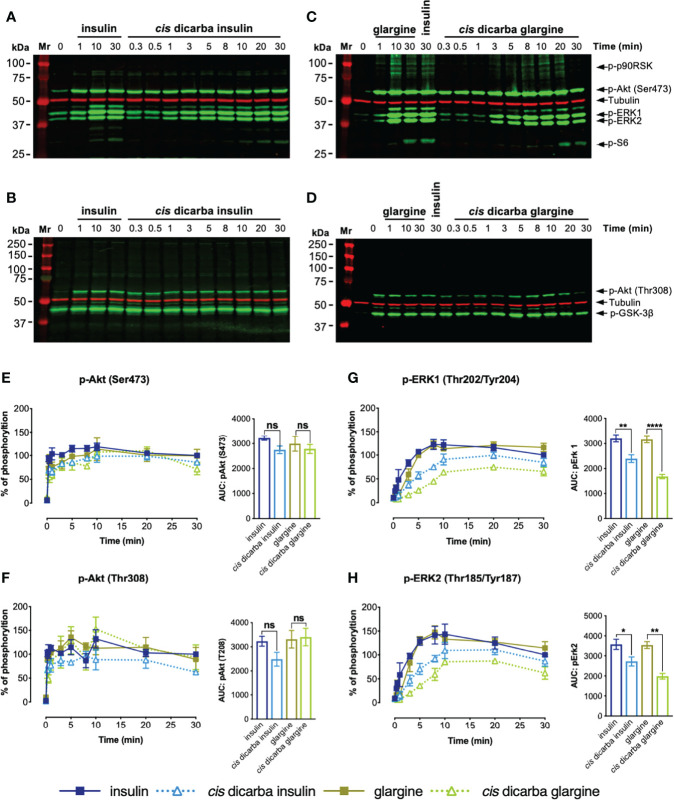
*Cis-*dicarba insulin and *cis-*dicarba glargine insulin exhibit time-dependent signaling bias *via* IR-A overexpressing cells. Serum starved IR-A overexpressing L6 myoblasts (hIR-A L6) were stimulated with 10 nM of **(A, B)** insulin or *cis-*dicarba insulin or **(C, D)** glargine or *cis-*dicarba glargine in a time-course of *t* = 0, 0.33 (20 s), 0.5 (30 s), 1, 3, 5, 8, 10, 20 and 30 min. The full time-course of insulin and glargine can be seen in **Figure S6**. **(E-H)**: Quantitation of western blots (n = at least 3 independent experiments). Phosphorylation levels are expressed as percentage of level detected when cells were stimulated with 10 nM of insulin for 30 min; normalized to β-tubulin: **(E)** Akt (phosho-Ser473), **(F)** Akt (phosho-Thr308), **(G)** ERK 1 (phospho-Thr202/Tyr204) and **(H)** ERK2 (phospho-Thr185/Tyr187). All data are the mean ± S.E.M. Error bars are shown when greater than the size of the symbols. AUC, area under the curves derived from % of phosphorylation over 30 minutes. Statistical significance of the difference in total phosphorylation over 30 minutes measured as AUC when stimulated by *cis-*dicarba insulin compared to insulin or *cis-*dicarba glargine compared to glargine insulin were determined *via* ordinary one-way ANOVA. ns: non-significant; * (P ≤ 0.05), ** (P ≤ 0.01); *** (P ≤ 0.001); **** (P ≤ 0.0001). Quantitation of other phosphorylated proteins are included in **Figure S7**.

Following the investigation of the dose-dependent response of *cis-*dicarba analogues, the kinetics of PI3K/Akt and MAPK signalling were determined. Intrigued by the apparent dose-dependent signalling bias driven under IR-A activation, we further investigated the signalling kinetics of stimulated L6 IR-A cells by insulin, *cis-*dicarba insulin, glargine and *cis-*dicarba glargine using a constant 10 nM concentration of ligand over a time course of t = 0, 0.33 (20s), 0.5 (30s), 1, 3, 5, 8, 10, 20 and 30 min (**Figures 5**; **S6, S7**). The overall kinetics of activation of the PI3K/Akt pathway (**Figures 5A-F**) versus the MAPK pathway (**Figures 5A, C, G, H**) by both insulin and glargine were markedly different. Akt (Ser473; **Figure 5E** and Thr308; **Figure 5F**) and GSK-3β (**Figure S7A**) showed a rapid response reaching optimal phosphorylation within 20s post-stimulation and remained at maximal phosphorylation throughout the duration of experiment up to t = 30 min. On the other hand, phosphorylation of ERK1/2 (**Figures 5G, H**) and p90RSK (**Figure S7B**) progressed gradually, reaching maximal phosphorylation at t = 10 min post-stimulation and then reducing between t = 10 – 30 min.

In line with the dose-response assays, the kinetics of activation of the PI3K/Akt pathway (p-Akt (Ser473; **Figure 5E**), p-Akt (Thr308; **Figure 5F**), p-GSK3β (Ser9; **Figure S7A**) and p-S6 (Ser235/236; **Figure S7C**)) were essentially the same for insulin, *cis-*dicarba insulin and *cis-*dicarba glargine. Downstream of Akt, the dicarba analogues were equipotent to their parent peptides in activating the S6 ribosomal protein, with all promoting gradual kinetics of activation, contrast to the rapid kinetics seen in other PI3K/Akt signalling proteins. These observations are consistent with the ability of *cis-*dicarba analogues and insulin to promote a similar level of metabolic activity *in vitro* (glucose uptake assays; **Figure 3A**) and *in vivo* (insulin tolerance tests; **Figure 3C**).

Compared to the equipotent insulin and glargine, a lower level of activation of MAPK signalling by *cis-*dicarba insulin and *cis-*dicarba glargine was characterized by more gradual ERK1/2 and p90RSK phosphorylation responses. The effect was more prominent for ERK 1/2 activation by *cis-*dicarba glargine (**p ≤ 0.01 to ****p ≤ 0.0001) than *cis-*dicarba insulin (*p ≤ 0.05 to **p ≤ 0.01) (**Figures 5G, H**). Moreover, when activated by *cis-*dicarba insulin and *cis-*dicarba glargine, ERK 1/2 phosphorylation never achieved the maximal level attained through stimulation by their corresponding parent peptides (insulin and glargine, respectively) (**Figures 5G, H**). These findings correlate well with the lower mitogenic potency of *cis-*dicarba analogues measured *in vitro via* the DNA synthesis assay (**Figure 3B**) and provide an explanation as to why metabolic potency is retained but mitogenic potency is reduced.

### IR-A Receptor Internalization

Having established the correlation between biological outputs and their upstream intracellular signalling kinetics, we proposed that the reduced mitogenic signalling of *cis-*dicarba insulins is regulated by a receptor-dependent event that occurs after the initial receptor engagement. Previously, other groups (24, 35, 36) and our laboratory (25) provided evidence supporting the hypothesis that activation of mitogenic signalling *via* MAPK pathway is dependent on IR-A internalization, although the underlying mechanism(s) that triggers receptor internalization is not fully understood. Therefore, we sought to determine if a decreased receptor internalization was associated with the poorer mitogenic potencies of the *cis-*dicarba insulins. The method used in this study for measuring receptor internalization was optimized based on previous methods and performed in L6 IR-A cells (24, 25, 37).

**Figure 6:**
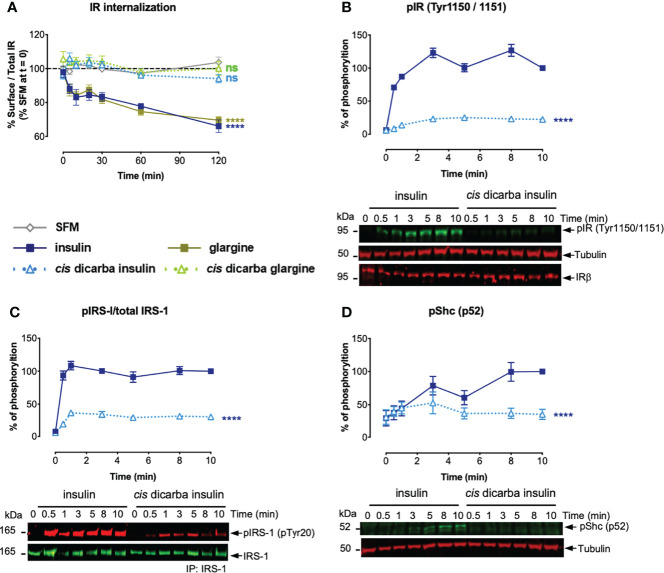
*Cis-*dicarba analogues are incapable of promoting IR-A internalization due to inability to promote phosphorylation of IR-A, IRS-1 and Shc. **(A)** Serum starved IR-A overexpressing L6 myoblasts were treated with serum-free media (SFM; non-stimulated condition, grey) or 10 nM of insulin analogues in a time-course of *t* = 0, 5, 10, 20, 30, 60 and 120 min. Data are presented as % of surface receptor/total receptor followed by normalisation with % of surface receptor in SFM at t = 0; i.e. SFM at *t* = 0 is equivalent to 100%. **(B, C)** Serum starved IR-A overexpressing L6 myoblasts (hIR-A L6) were stimulated with 10 nM of insulin or *cis-*dicarba insulin in a time-course of *t* = 0, 0.5 (30 s), 1, 3, 5, 8 and 10 min. Phosphorylation levels are expressed as percentage of level detected when cells were stimulated with 10 nM of insulin for 10 min: **(B)** IR (phospho-Tyr1150/Tyr1151); normalized to β-tubulin, IRβ for loading control C) immunoprecipitated (IP) IRS-1 (pTyr20: total tyrosine phosphorylation); normalized to total IRS-1 and **(D)** p52 Shc (phospho-Tyr239/Tyr240); normalized to β-tubulin. All data are the mean ± S.E.M. n = at least 3 independent experiments. Error bars are shown when greater than the size of the symbols. Statistical significance were determined *via* one-way repeated measures ANOVA followed by Dunnets multiple test. For receptor internalization **(A)**, statistical analyses were performed comparing the effect of insulin analogues and SFM. For **(B-D)**, statistical analyses were performed comparing insulin and *cis-*dicarba insulin. ns: non-significant; **** (P ≤ 0.0001).

Insulin and glargine rapidly induced receptor internalization post-stimulation (evident within 10 min). Within 2 h, only 60% of IR-A remained on the cell surface (**Figure 6A**). As predicted, the less mitogenic *cis-*dicarba insulin and *cis-*dicarba glargine showed significantly impaired ability to promote IR-A internalization, with virtually all receptor remaining on the surface 2 hours post-stimulation.

### Kinetics of IR-A, IRS-1 and Shc Activation

To determine whether reduced IR-A internalization in response to *cis-*dicarba insulins was a result of perturbation of the initial receptor activation step, the phosphorylation of Tyr1150/Tyr1151 (Tyr residues located within the kinase domain activation loop) was measured upon *cis-*dicarba insulin activation (**Figure 6B**). While there was rapid and sustained phosphorylation over 10 min of Tyr1150/Tyr1151 in response to insulin, the *cis-*dicarba insulin was much slower to stimulate receptor activation and the response only reached ~20% that of insulin. This result was unexpected as *cis-*dicarba insulin was almost as potent as insulin in a kinase receptor activation assay in which the phosphorylation of tyrosine on immunocaptured, activated receptors is measured (9), and serves as a reminder that the KIRA assay is a read out of total receptor phosphorylation and not specifically the phosphorylation of the kinase activation loop. In turn, the ability of *cis-*dicarba insulin to promote IRS-1 (**Figure 6C**) and Shc (**Figure 6D**) activation was equally perturbed resulting in much slower and weaker responses (~30%) compared to insulin. As *cis-*dicarba insulin and *cis-*dicarba glargine were equally ineffective in promoting IR-A internalization we assume similar effects on Tyr1150/Tyr1151 phosphorylation and IRS-1 and Shc activation would be seen for *cis-*dicarba glargine. Overall these results indicate that IR-A internalisation is dependent on effective IRS-1 and Shc activation in order to promote effective ERK1/2 activation.

## Discussion

In the last three decades many insulin analogues have been generated to provide effective options for the treatment of diabetes. The early discovery that insulin X10 had increased mitogenic properties highlighted the need to be aware of the potential to introduce unwanted mitogenic properties into newly designed analogues. We aimed to develop a glargine analogue with reduced mitogenic potency through the introduction of a dicarba bond at residues A6-A11. The resultant *cis-*dicarba glargine binds the IR with equal potency to insulin, promotes equivalent glucose uptake *in vitro* and *in vivo* and yet has marked reduction in mitogenic potency (DNA synthesis). As seen with our previous studies with dicarba insulin, the *trans-*dicarba glargine isomer was unable to bind the IR and was therefore inactive.

**Figure 7:**
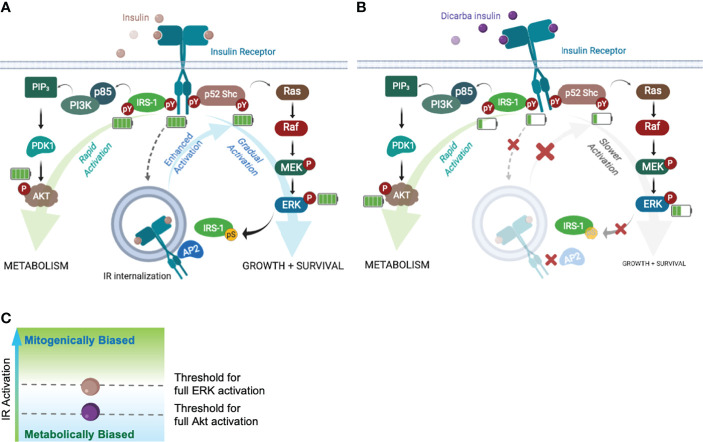
Akt and ERK requires different IR activation thresholds to achieve full activation that influence the fate of biological outputs. Schematic of the Akt-PI3K and ERK-MAPK signalling pathways following ligand binding. Increased/decreased green bars in battery symbol represent an increase or decrease in levels of phosphorylation. **(A)** Insulin binding to the IR with full activation of pathways leading to metabolism, growth and survival. **(B)** Dicarba insulin binding to the IR with decreased activation of the IR, IRS-1, Shc and ERK1/2 resulting in impaired feedback to IRS-1 and decreased IR internalization (decreased growth and survival). Akt signalling continues equivalent to the response to insulin **(C)** Schematic indicating the threshold levels of IR activation that are required for full ERK and/or full Akt activation (mitogenically or metabolically biased).

To explain how *cis-*dicarba glargine acts as a biased IR agonist we investigated the signalling steps leading to the different biological outcomes. Initially the ability to activate Akt and ERK1/2 was monitored as an indication of ability to regulate metabolic and mitogenic responses, respectively. *Cis-*dicarba glargine stimulated rapid activation of Akt with similar rapid kinetics and potency to insulin, whereas it was less potent in promoting the slower kinetics of ERK1/2 activation than insulin (**Figure 5**). The slower kinetics and lower potency of *cis-*dicarba glargine were similar to those of *cis-*dicarba insulin, suggesting that the *cis-*dicarba bond properties are conferring an altered ability to activate the ERK1/2 pathway. Here we also show that *cis-*dicarba insulin and *cis-*dicarba glargine were ineffective in promoting IR internalisation (**Figure 6A**), a property that has previously been shown to be required for effective and potent ERK1/2 activation (25, 26, 38). Notably, internalisation involves feedback of ERK1/2 to phosphorylate IRS-1, which allows recruitment of assembly polypeptide 2 (AP2) and the endocytic machinery (see **Figure 7**) (39). This requirement for feedback likely underlies the relatively gradual phosphorylation kinetics of ERK1/2 (40). It is possible that *cis-*dicarba glargine and *cis-*dicarba insulin are ineffective in promoting the required feedback and subsequent recruitment of AP2 leading to ineffective IR internalisation and resulting in much reduced ERK1/2 activation compared to insulin (**Figure 7B**).

To explain why both dicarba insulins (*cis-*dicarba glargine and *cis-*dicarba insulin) were equipotent to insulin in activating Akt, and yet both were ineffective in stimulating internalization and ERK1/2 activation, we speculated that signalling upstream of Erk1/2 was affected. To explore this, we measured the ability of *cis-*dicarba insulin to activate the IR-A (phosphorylation of Tyr1150/Tyr1151 of the tyrosine kinase activation loop, IR-A numbering) and discovered reduced kinetics of activation compared to insulin. This is similar to the poorly mitogenic S597 peptide, which also promoted slower IR-A Tyr phosphorylation kinetics compared to insulin, not only for Tyr residues on the activation loop (Tyr1146, Tyr1150, Tyr1151) but also Tyr960 (in the Juxtamembrane domain, a docking site for the adaptor proteins IRS 1/2 and p52 Shc) and several residues on the C-terminal domain (Tyr1316 and Tyr1322) (25, 26). In contrast, the mitogenic insulin X10 promoted an increased phosphorylation of IR-A Tyr960, Tyr1146 and Tyr1322 compared to insulin (32), with a small preferential phosphorylation of Tyr960 and Tyr1146 over the C-terminal Tyr1322. Therefore, this study provides further evidence that the magnitude and kinetics of IR Tyr phosphorylation play a key role in determining the mitogenic potency of insulin analogues.

Next we measured the phosphorylation of the adapter proteins IRS-1 and Shc (**Figures 6C, D**). Recruitment and rapid activation of IRS 1/2 and p52 Shc adaptor proteins leads to Akt and MAPK pathway activation (**Figure 7A**), respectively, and we wondered if a difference in their activation could explain the bias to Akt signalling. We showed that *cis-*dicarba insulin was significantly less potent in activating both IRS-1 and Shc, in line with the decreased IR phosphorylation. To reconcile how Akt signalling promoted by *cis-*dicarba insulin remains equal to insulin despite poor receptor activation, we propose that IR signalling bias is modulated through different IR phosphorylation thresholds (**Figure 7C**). We propose that full activation of Akt is rapid and can be achieved *via* a low level of IR Tyr1150/Tyr1151 phosphorylation and IRS-1 activation. Full activation of ERK1/2 only occurs when Tyr1150/Tyr1151 phosphorylation and Shc activation exceeds a higher threshold that permits sufficient ERK1/2 feedback to IRS-1 to recruit AP2 and promote internalisation. A similar proposal has been put forward to explain the bias of the anti-IR monoclonal antibody XMetA which effectively activates the Akt pathway but fails to activate ERK (27). Such a mechanism has been previously described for the fibroblast growth factor receptor (FGFR), where the durability/strength of FGFR dimer formation promoted by ligand binding governs different levels of receptor activation and downstream signalling (41).

The evidence provided so far suggests that installation of an intrachain dicarba bridge in insulin and glargine affects the way in which the analogues engage with and subsequently activate the IR. A key structural change in insulin results in the end of the B-chain opening out from the insulin core to enable important residues at the beginning of the first A-chain to bind to the αCT’ within the IR binding site 1. Previously we conducted a detailed biophysical analysis of both the *cis-*and *trans-*dicarba isomers to explain why the *trans*-isomer is inactive (30). Both *cis*- and *trans*-dicarba bonds are metabolically non-reducible, planar and conformationally restricted. We showed that a *cis*-configured dicarba A6-A11 bridge permits A-chain flexibility which is apparently important for the transition of insulin to its active conformation for IR binding. Molecular dynamics simulations suggested that the short A6-A11 Cα-Cα distance in *cis*-dicarba bond allows the active conformation of the A chain to be accessed. The *trans*-dicarba A6-A11 bond has decreased conformational flexibility and a longer A6-A11 Cα-Cα distance changing the conformation of the A6-A11 loop, thereby preventing the formation of a hydrogen bond between ThrA8 and ValA3 in the A-chain helix and inhibiting it from adopting the active conformation that engages with binding site 1.

Recent cryo-electron microscopy studies of the IR have captured the structure of the insulin:IR complex in what is believed to be the active conformation (13, 15). A major rearrangement of the IR involves movement of the Fn-III-1-3 “legs” together which apparently brings the transmembrane spanning regions in close proximity and leads to activation of the intracellular tyrosine kinase domain (although the intracellular region is not resolved in the structures). In the active conformation the binding site 1 interaction involves the L1, αCT’ and less extensive contact with the Fn-III-1’ (termed site 1b’ (15),). Of note is that insulin’s A6-A11 loop and surrounding residues including the interchain CysA7-CysB7 disulfide bond are in close proximity to site 1b’. It may be possible that not only does the A6-A11 Cα-Cα distance and the lack of flexibility play a role in engagement with the αCT’ but it can also impact the site 1b’ interaction through the impact on the A6-A11 loop and CysA7-CysB7 bond. Interestingly, a key residue in the site 1b’ interaction is insulin HisB10 which lies near CysB7 and contacts IR Arg539’in the final active conformation. Substitution of HisB10 with Asp leads to an increased rate of receptor internalisation and enhanced mitogenic signalling that is related to its slower kinetics of dissociation from the IR (20). It is tempting to speculate that the lower mitogenic potential of *cis-*dicarba insulins, including *cis-*dicarba glargine, is due to a change in the interaction at site 1b’ through altering the flexibility of insulin in this region and more specifically impacting the CysA7-CysB7 bond. Implied is then that the *cis-*dicarba insulins would have an increased rate of dissociation through altered interaction at this site and this would lead to the reduced IR activation, IR internalisation and ERK1/2 signalling. It is less likely that the effect of the *cis-*dicarba bond on the A6-A11 loop and CysA7-CysB7 bond would impact on binding at site 2 on Fn-III-1’ (residues Arg479, Ser481, Lys484, Leu486, Arg488, Asp535, Pro537, Pro549, Gly550, Trp551, Leu552 (15)), despite HisB10 also being involved in site 2 binding. The A6-A11 loop is on the opposite side of insulin to the site 2 binding region and HisB10 is peripheral to this binding site (15–17). However, a bulge introduced in the *cis*-dicarba insulin B chain close to site 2 residue LeuB17 may impact site 2 binding (30). Study of *cis-*dicarba dissociation kinetics and determination of a structure of the dicarba insulin: IR-A complex would reveal any impact on the Fn-III-1’ interaction and is in future plans.

In conclusion, through minimal manipulation (introduction of a non-reducible A6-A11 dicarba bridge) of insulin, we have created ideal analogues (*cis-*dicarba insulin and *cis-*dicarba glargine) for the investigation of IR-A signalling bias. Analysis of the receptor binding, activation and downstream signalling confirmed that the activation of MAPK signalling *via* IR-A is dependent on the rate of IR-A internalization. We conclude that replacement of the A6-A11 disulfide bond with a *cis*-configured, rigid dicarba bond does not affect metabolic potency but significantly reduces mitogenic potential. It appears that the ability to reach an IR activation threshold that permits ERK1/2 activation is dependent on interaction between the insulin structure at the A6-A11 loop and the residues in close proximity to the CysA7-CysB7 bond and the IR Fn-III-1’, possibly at site 1b. This advance in our understanding of molecular determinants of IR activation will be important for the future design of improved insulin analogues which are metabolically effective and with minimal mitogenic activity.

## Experimental Procedures

### Materials

Actrapid^®^ insulin was purchased from Novo Nordisk Pharmaceuticals Ltd. Lantus^®^ glargine insulin was purchased from Sanofi Pty Ltd. Western Lightning Plus-ECL, Enhanced Chemiluminescence Substate was purchased from Perkin Elmer, Amersham™ Protran™ 0.2 μm nitrocellulose membrane was obtained from GE Healthcare Life Sciences. Precision Plus Protein™ Dual Color Standards was purchased from Bio-Rad. Hybridoma cells expressing antibodies specific for the IR α-subunit (83-7 and 83-14), IGF-1R α-subunit (24-31) and IR β-subunit (CT-1) were a gift from Prof. K Siddle (42–45). The monoclonal anti-IR antibody 83-14 was labelled with europium (Eu-83-14) according to instructions provided by Perkin Elmer Life Sciences. Anti-Eu PY20 was purchased from Perkin Elmer Life Sciences. 5-Ethynyl-2’-deoxyuridine (EdU) was purchased from Abcam, and FAM-Azide 488 was purchased from Lumiprobe Corporation. Blots were probed with Pathscan^®^ Multiplex Western Cocktail (Cell Signalling, #5301S, 1:200) to detect phospho-p90RSK (Ser380), phospho-Akt (Ser473), phospho-ERK1 (Thr202/Tyr204), phospho-ERK2 (Thr185/Tyr187) and phospho-S6 ribosomal (Ser235/236) simultaneously or with antibodies against phospho-AS160 (Thr642, 1:1000), phospho-Akt (Thr308, 1:1000) and phospho-GSK3b (Ser9, 1:1000) (Cell Signalling). Phospho IR (Tyr1151/Tyr1150, 1:1000) and phospho Shc (Tyr239/Tyr240, 1:1000) were from Cell Signalling, anti-β tubulin (1:1000) was purchased from Thermo Fisher Scientific, pTyr20 (1:200) was from Santa Cruz and IRS-1 antibody (1:500) is from Merck. The IR β antibody (1:400 for western blots) was a gift from Prof. K Siddle. Secondary antibodies (donkey anti-mouse IgG IR Dye 680RD and donkey anti-rabbit IgG IR Dye 800CW, 1:50,000) were purchased from LI-COR, (goat anti-mouse IgG HRP conjugated, 1:5000) from Thermo Fisher and (donkey anti-rabbit IgG HRP conjugated, 1:5000) from Jackson Immunoresearch.

## Cell Lines and Culture Conditions

Human IR-A and IR-B over-expressing R^-^ fibroblast cells (R^-^IR-A and R-IR-B, respectively) derived from IGF-1R knockout mouse embryonic fibroblasts, a gift from Prof. R. Baserga (Philadelphia, USA) (46) were produced according to Denley et al. (33). Human IR-A over expressing L6 myoblasts (L6 IR-A cells) were provided by Dr B.F. Hansen (Novo Nordisk A/S, Denmark). P6 cells (BALB/c3T3 cells overexpressing the human IGF-1R) were from Prof. R. Baserga (47). All cells were maintained in Dulbecco’s minimal essential medium (DMEM) high glucose (4500 mg/L) supplemented with 10% FCS (Bovogen Biologicals), 100 U/L penicillin and 100 µg/L streptomycin. Cell culture media and supplements were purchased from Life Technologies/Thermo Fisher Scientific Australia.

### Synthesis of Dicarba Glargine Insulins

Chemical synthesis of dicarba insulins was performed as previously described (9), the synthesis of dicarba glargine insulins is outlined in **Figure S1**. The methods of synthesis of c[Δ^4^A6,11]-dicarba insulin (*cis*- and *trans-*dicarba insulins) (9) and c[Δ^4^A6,11]-dicarba insulin glargine (*cis*- and *trans-*dicarba glargines) were essentially the same. Synthesis of dicarba A chain was achieved through an interrupted solid-phase peptide synthesis (SPPS)-catalysis and ring-closing metathesis (RCM) procedures (9, 31). Construction of glargine insulin A- and B-chain was achieved through microwave-accelerated SPPS. The monocyclic A-B conjugates were prepared by combination of the dicarba glargine A chain with the glargine insulin B chain under basic conditions resulting in spontaneous oxidation of the liberated free thiol groups. This was followed by iodine induced concurrent CysA19 and CysB20 deprotection and oxidation generating the target *cis*- and *trans*-dicarba glargine insulins.

### Receptor Competition Binding Assays

IR-A, IR-B and IGF-1R competition binding with increasing concentrations of insulin, glargine insulin and *cis-*dicarba insulins was measured as previously described by Denley et al. (33) and as reported in Ong et al. (30). Human IR isoform A, isoform B and IGF-1R were solubilized from R^-^IR-A, R^-^IR-B and P6 cells, respectively. Briefly, cells were serum starved in serum-free medium (SFM) containing 1% (w/v) BSA for 4 h before lysis in ice-cold lysis buffer (20 mM HEPES, 150 mM NaCl, 1.5mM MgCl_2_, 10% (v/v) glycerol, 1% (v/v) Triton X-100, 1mM EGTA, and 1 mM phenylmethylsulfonyl fluoride, pH 7.5) for 1 h at 4°C. Lysates were centrifuged for 10 min at 2,200g, and then 100 µL of lysate was added per well to a white Greiner Lumitrac 600 96-well plate (16 h at 4°C) previously coated with anti-IR antibody 83-7 or anti-IGF-1R antibody 24-31 (250 ng/well in bicarbonate buffer, pH 9.2). Approximately 300-500,000 fluorescent counts of europium-labelled insulin or IGF-I (Eu-insulin or Eu-IGF-I prepared in house) were added to each well along with increasing concentrations of unlabelled competitor and incubated for 16 h at 4°C. Wells were washed with 20 mM Tris, pH 7.4, 150 mM NaCl (TBS) and 0.1% (v/v) Tween 20 (TBST). Then 100 µl/well DELFIA enhancement solution (PerkinElmer Life Sciences) was added. After 10 min, time-resolved fluorescence was measured using 340-nm excitation and 612-nm emission filters with a BMG Lab Technologies Polarstar fluorometer (Mornington, Australia). Assays were performed in triplicate in at least three independent experiments.

### Kinase Receptor Activation Assays (KIRA)

IR-A, IR-B and IGF-1R phosphorylation in response to increasing concentrations of insulin, glargine insulin and *cis-*dicarba insulins was detected essentially as previously described (30, 33). Briefly, R^-^IR-A, R^-^IR-B or P6 cells (5x10^4^ cells/well) were serum-starved for 4 h before treatment with increasing concentrations of insulin, glargine or *cis-*dicarba insulins (0.01 - 300 nM) in 100 µl of SFM/1% BSA for 10 min or over a time course with 10 nM ligand (0, 2, 5, 8, 12, 20, and 30 min) at 37°C/5% CO_2_. Cells were lysed with ice-cold lysis buffer containing 2 mM Na_3_VO_4_ and 100 mM NaF, and receptors were captured onto white Greiner Lumitrac 600 96-well plates precoated with anti-IR antibody 83-7 or anti-IGF-IR antibody 24-31 (250 ng/well) and blocked with TBST/0.5% BSA. Following overnight incubation at 4°C, the plates were washed three times with TBST. Phosphorylated receptor was detected by incubation with Eu-PY20 (76 ng/well) at room temperature for 2 h. Wells were washed four times with TBST, and time-resolved fluorescence was detected as described above. Assays were performed in triplicate in at least three independent experiments.

### DNA Synthesis Assay

DNA synthesis was carried out based on previously described Gaugin et al. (48), with some modifications using the labelling approach described below. L6 rat skeletal myoblasts overexpressing the human IR-A were used as they are a well-established model for measuring mitogenic activity of insulin analogues (49). These cells express 287,000 IR-A receptors compared to 25,800 IGF-1R receptors (50), meaning the IR-A homodimer is the predominant receptor in this cell line and the responses reflect mitogenic activity promoted by IR-A. Cells were plated in a 96 well flat bottom plate (32 × 10^4^ cells/well) and grown overnight at 37°C, 5% CO_2_. Cells were starved in SFM for 2 h prior to treatment with increasing ligand concentrations of insulin, glargine or *cis-*dicarba insulins (0.01 - 300 nM) for 18 h in SFM/1% BSA at 37°C/5% CO_2_. The cells were incubated with 10 µM of 5-Ethynyl-2’-deoxyuridine (EdU) for 4 h, washed with filtered PBS/1% BSA and fixed in the dark for 15 min with 4% paraformaldehyde (PFA). Fixed cells were washed with PBS/1% BSA and permeabilized for 20 min with 0.5% Triton X-100. A click chemistry labelling cocktail (2 µM FAM-Azide 488/100 mM Tris pH 7.5/4 mM CuSO_4_/100 mM sodium ascorbate) was added to the cells for 30 min at room temperature in the dark. Finally, cells were washed thrice with PBS/1% BSA and fluorescence was measured using 485 nm excitation and 535 nm emission filters with the BMG Lab Technologies Polarstar fluorometer. Assays were performed in triplicate in at least three independent experiments.

### Glucose Uptake Assay

NIH3T3-L1 myoblasts (up to passage 20) grown in DMEM supplemented with 10% newborn calf serum, 2mM L-glutamine, 100 U/l penicillin, 100 µg/L streptomycin at 37°C were seeded into 24-well plates at 5 x 10^3^ cells/well and grown for 8 days to confluence. They were then differentiated into adipocytes as described in Govers *et* al. (51). After differentiation the NIH3T3-L1 cells express 37,000 IGF-1R and 250,000 IR per cell (52). Glucose uptake in response to insulin and insulin analogues was measured essentially as described in van Dam et al. (53). Briefly, 3T3-L1 adipocytes were serum starved in SFM/1% BSA for 4 h, washed twice with Krebs-Ringer phosphate buffer (KRP; 12.5mM HEPES, 120mM NaCl, 6mM KCl, 1.2mM MgSO_4_, 1mM CaCl_2_, 0.4mM Na_2_HPO_4_, 0.6mM Na_2_HPO_4_ (pH7.4)) containing 1% BSA and incubated for 15min at 37°C. Insulin or insulin analogues were added at decreasing concentrations (100 – 0.3nM) for 30 min at 37°C. For the final 10 min 2-deoxyglucose (DOG) uptake was initiated by the addition of 50 µM cold deoxyglucose and 1 µCi ^3^H –deoxyglucose per well. The assay was terminated by rapidly washing the cells three times with ice-cold KRP buffer. Cells were solubilized in 0.5 M NaOH/0.1% SDS and ^3^H content was determined by scintillation counting. Nonspecific 2-DOG uptake was determined in the presence of 50μM cytochalasin B. Assays were performed in triplicate in at least three independent experiments.

### Insulin Tolerance Test

Insulin tolerance tests were performed as previously described (9). Briefly, eight-week-old C57BL6 male mice were fed a standard chow diet containing (wt/wt) 77% carbohydrate, 20% protein and 3% fat from Ridley AgriProducts (Pakenham, Victoria, Australia). Mice (6 per group) were injected ip with 0.75I U/Kg insulin, glargine, *cis-*dicarba insulin or *cis-*dicarba glargine under non-fasting conditions and tail vein blood glucose was monitored using a glucometer at indicated times. Experimental procedures were carried out in accordance with protocols approved by Austin Health Animal Ethics Committee (AEC 2011/04396).

### Immunoprecipitation and Western Blot Analysis

R/^-^IR-B (80,000 cells/well) or L6 IR-A cells (240,000 cells/well) were seeded in 6-well plates and allowed to grow to confluence (~ 48 hours). Prior to stimulation, cells were serum starved in SFM/1% BSA for 4 h. Cells were stimulated and analysed by dose-response (0.1, 1, 10 and 100 nM) for 10 min at 37°C and by time-course at 10 nM ligand concentration at 37°C (stimulating for various times up to 30 min) with insulin or insulin analogues. After stimulation, cells were washed with warm PBS and lysed for 60 min in 200 μL pre-chilled RIPA lysis buffer (50 mM Tris, 150 mM NaCl, 1% (v/v) NP-40, 0.25% sodium deoxycholate, 1 mM EDTA, pH 8.0) supplemented with Roche cOmplete™ Protease Inhibitor Cocktail and PhosSTOP™ phosphatase inhibitor tablets. Lysates were scraped from the well, spun at 13,000 rpm for 3 min at 0°C to remove cell debris and stored at -80°C. Lysate protein concentrations were quantified using Bio-Rad DC™ Protein Assay and 20μg of each sample was separated in 10% glycine gels under reducing conditions. Transferred blots were blocked for 1 h at room temperature in TBST containing 3% BSA and probed with primary antibodies (Pathscan Antibody, pAkt(Thr308), pGSK(Ser9), pAS160(Thr642)) in blocking solution (TBST containing 3% BSA) overnight at 4°C. All blots were also probed with anti-β-tubulin as a loading control. Blots were washed six times for 5 min each in TBST then probed with either HRP-conjugated (dose-response) or fluorophore-conjugated (time-course) secondary antibodies for 1 hour at room temperature. The washing was then repeated.

Activated IRS-1 was determined using immunoprecipitation. Anti-IRS-1 antibody(4ug) was pre incubated with protein G agarose overnight at 4°C before adding to lysates to immunoprecipitate IRS-1 overnight at 4°C. Complexes were washed three times in PBS, boiled under reducing conditions and samples separated on 10% glycine gels. Transferred blots were blocked for 1 h at room temperature in TBST containing 3% BSA and probed with primary antibodies (anti-IRS-1 for total IRS-1 protein, or anti pTyr20 for total phosphorylated protein in immunoprecipitation) in blocking solution (TBST containing 3% BSA) overnight at 4°C. Blots were washed six times for 5 min each in TBST then probed with fluorophore-conjugated secondary antibodies (donkey anti-mouse 680RD and anti- rabbit 800CW) for 1 hour at room temperature. The washing was then repeated before blots were imaged with an Odyssey^®^ CLx Imaging System.

Dose response blots were developed in ECL reagent according to manufacturer’s instruction and imaged in Fujiflim LAS-4000 Luminescent Image Analyzer. Time course blots were imaged with an Odyssey^®^ CLx Imaging System. All blots were performed at least three times. All data were analysed with LI-COR Image Studio™ software and each band intensity was normalized to tubulin of its respective lane. Densitometric analyses were performed to demonstrate dose-response (Equation 1) and time-course (Equation 2) effects of *cis-*dicarba insulin analogues on phosphorylation levels of intracellular proteins involve in metabolic and (or) mitogenic signalling.

Equation 1: Dose-response Analysis


$$\%ofphosphorylation\left(overphosphorylationlevelstimulatedby100nMinsulin\right)=\frac{\mathrm{Normalised}\mathrm{signal}\mathrm{stimulated}\mathrm{by}x\mathrm{nM}\mathrm{insulin}/\mathrm{analogue}}{\mathrm{Normalised}\mathrm{signal}\mathrm{stimulated}\mathrm{by}100\mathrm{nM}\mathrm{insulin}}x100\%$$

Data were analysed individually, averaged and plotted as mean ± S.E.M. in bar graphs.

Equation 2: Time-course Analysis


$$\%ofphosphorylation\left(overfinaltimepointinsulinstimulation\right)=\frac{\mathrm{Normalised}\mathrm{signal}\mathrm{stimulated}\mathrm{by}10\mathrm{nM}\mathrm{insulin}/\mathrm{analogue}\mathrm{at}t=x\min}{\mathrm{Normalised}\mathrm{signal}\mathrm{stimulated}\mathrm{by}10\mathrm{nM}\mathrm{insulin}\mathrm{at}t=\mathrm{final}\mathrm{time}\mathrm{point}}x100\%$$

Data were analysed individually, averaged and plotted as mean ± S.E.M. in XY line graphs.

### Receptor Internalization

Receptor internalization assays were performed based on methods described previously with some modifications (24, 25, 37). Briefly, R-IR-A cells (10,000 cells/well) were seeded in 96-well plates in duplicate sets: one to measure surface receptor after stimulation (surface receptor plate); the other to measure total receptors (total receptor plate). Cells were allowed to grow for ~ 48 h to confluence. Prior to stimulation, cells were serum starved in SFM/1% BSA for 4 h. Time-course analyses were performed with 10 nM ligand concentration stimulating for t = 0, 5, 10, 20, 30, 60 and 120 min. After stimulation, medium was aspirated, and cells in the surface receptor plate were fixed with 4% paraformaldehyde/PBS for 15 min and washed thrice with TBS. Plates were blocked overnight with TBS containing 1% BSA. The cells in the separate total receptor plate were lysed in 110 µL ice-cold RIPA lysis buffer and incubated for 1 h, at 4 °C. Receptors were captured in white Greiner Lumitrac 600 96-well plates pre-coated with anti-IR antibody 83-7 (250 ng/well). Following overnight incubation at 4°C, both plates were washed three times with TBST. Approximately 500,000 fluorescent counts of Eu-83-14 antibody were added to each well diluted with europium binding buffer (100 mM HEPES, 100 mM NaCl, 0.5% Tween-20, 2 μM DTPA, pH 8.0) in final volume of 100 µL/well and incubated in the dark for 1 h. Wells were washed three times with TBST. DELFIA enhancement solution was added into both plates (100 µL/well). After 10 min, the solutions in the surface receptor plate were transferred to a white Greiner Lumitrac 600 96-well plate. Finally, time-resolved fluorescence was measured using 340 nm excitation and 612 nm emission filters with a BMG Lab Technologies Polarstar fluorometer (Mornington, Australia). Assays were performed in triplicate in at least three independent experiments. The extent of receptor internalization upon ligand stimulation at each time-point was analysed using Equation 3 followed by normalization with % surface receptor under non-stimulated conditions (SFM) at t = 0.

Equation 3:


$$\%\;ofSurface/Total\;Receptor\;=\frac{Surface\;Receptor\;at\;T=x}{Average\;Total\;Receptor}x100\%$$

Data were analysed individually, averaged and plotted as mean ± S.E.M. in XY line graphs.

### Statistical Analyses

Statistical analysis of receptor binding, receptor activation and DNA synthesis assays were performed using a 2-way ANOVA with a Dunnett’s multiple comparison. Data for glucose uptake assay and significance of the overall change of blood glucose levels in insulin tolerance tests were analysed by paired t-test. Significance of the change of blood glucose levels at each time-point was also determined by 2-way ANOVA followed by Holm Sidak’s multiple comparison test. Significance was accepted at P < 0.05.

## Data Availability Statement

The original contributions presented in the study are included in the article/**Supplementary Material**. Further inquiries can be directed to the corresponding author.

## Ethics Statement

The animal study was reviewed and approved by Austin Health Animal Ethics Committee, Melbourne.

## Author Contributions

SC performed the majority of *in vitro* activity assays, AM performed the DNA synthesis and IR-A receptor binding assays, CAD performed immunoblotting. AB and BL synthesized dicarba insulins. *In vivo* experiments were conducted in the lab of SA. The study was conceived and planned by BEF and AR. SC, AM, and BF wrote the manuscript. All authors reviewed and approved the final version of this paper.

## Funding

We acknowledge the funding for this study from NHMRC (APP1069328) and ARC Linkage (LP120200792).

## Conflict of Interest

The authors declare that the research was conducted in the absence of any commercial or financial relationships that could be construed as a potential conflict of interest.

## Publisher’s Note

All claims expressed in this article are solely those of the authors and do not necessarily represent those of their affiliated organizations, or those of the publisher, the editors and the reviewers. Any product that may be evaluated in this article, or claim that may be made by its manufacturer, is not guaranteed or endorsed by the publisher.

## References

[1] Bonnesen C Nelander GM Hansen BF Jensen P Krabbe JS Jensen MB. Synchronization in G0/G1 Enhances the Mitogenic Response of Cells Overexpressing the Human Insulin Receptor A Isoform to Insulin. Cell Biol Toxicol (2010) 26:293–307. doi: 10.1007/s10565-009-9142-x 19898946 PMC2896650

[2] Janssen JA Varewijck AJ. Insulin Analogs and Cancer: A Note of Caution. Front Endocrinol (2014) 5:79. doi: 10.3389/fendo.2014.00079 PMC4033362 24904529

[3] Johnson JA Gale EA. Diabetes, Insulin Use, and Cancer Risk: Are Observational Studies Part of the Solution-or Part of the Problem? Diabetes (2010) 59:1129–31. doi: 10.2337/db10-0334 PMC2857892 20427699

[4] Tennagels N Werner U. The Metabolic and Mitogenic Properties of Basal Insulin Analogues. Arch Physiol Biochem (2013) 119:1–14. doi: 10.3109/13813455.2012.754474 23373726 PMC3581051

[5] Karlstad O Starup-Linde J Vestergaard P Hjellvik V Bazelier MT Schmidt MK. Use of Insulin and Insulin Analogs and Risk of Cancer - Systematic Review and Meta-Analysis of Observational Studies. Curr Drug Saf (2013) 8:333–48. doi: 10.2174/15680266113136660067 PMC3899599 24215311

[6] Vigneri P Frasca F Sciacca L Pandini G Vigneri R. Diabetes and Cancer. Endocr Relat Cancer (2009) 16:1103–23. doi: 10.1677/ERC-09-0087 19620249

[7] Sciacca L Vella V Frittitta L Tumminia A Manzella L Squatrito S. Long-Acting Insulin Analogs and Cancer. Nutr Metab Cardiovasc Dis (2018) 28:436–43. doi: 10.1016/j.numecd.2018.02.010 29609864

[8] Whittaker L Hao C Fu W Whittaker J. High-Affinity Insulin Binding: Insulin Interacts With Two Receptor Ligand Binding Sites. Biochemistry (2008) 47:12900–9. doi: 10.1021/bi801693h PMC2819479 18991400

[9] van Lierop B Ong SC Belgi A Delaine C Andrikopoulos S Haworth NL. Insulin in Motion: The A6-A11 Disulfide Bond Allosterically Modulates Structural Transitions Required for Insulin Activity. Sci Rep (2017) 7:17239. doi: 10.1038/s41598-017-16876-3 29222417 PMC5722942

[10] De Meyts P. Insulin/receptor Binding: The Last Piece of the Puzzle? What Recent Progress on the Structure of the Insulin/Receptor Complex Tells Us (or Not) About Negative Cooperativity and Activation. BioEssay News Rev Molecul Cell Dev Biol (2015) 37:389–97. doi: 10.1002/bies.201400190 25630923

[11] Menting JG Whittaker J Margetts MB Whittaker LJ Kong GK Smith BJ. How Insulin Engages its Primary Binding Site on the Insulin Receptor. Nature (2013) 493:241–5. doi: 10.1038/nature11781 PMC3793637 23302862

[12] Jensen M. Analysis of Structure-Activity Relationships at the Insulin Molecule by Alanine-Scanning Mutagenesis. Master Thesis Univ Copenhagen (2000).

[13] Weis F Menting JG Margetts MB Chan SJ Xu Y Tennagels N. The Signalling Conformation of the Insulin Receptor Ectodomain. Nat Commun (2018) 9:4420. doi: 10.1038/s41467-018-06826-6 30356040 PMC6200814

[14] Scapin G Dandey VP Zhang Z Prosise W Hruza A Kelly T. Structure of the Insulin Receptor-Insulin Complex by Single-Particle Cryo-EM Analysis. Nature (2018) 556:122–5. doi: 10.1038/nature26153 PMC5886813 29512653

[15] Uchikawa E Choi E Shang G Yu H Bai XC. Activation Mechanism of the Insulin Receptor Revealed by Cryo-EM Structure of the Fully Liganded Receptor-Ligand Complex. eLife (2019) 8:e48630. doi: 10.7554/eLife.48630 31436533 PMC6721835

[16] Gutmann T Schafer IB Poojari C Brankatschk B Vattulainen I Strauss M. Cryo-EM Structure of the Complete and Ligand-Saturated Insulin Receptor Ectodomain. J Cell Biol (2020) 219:e201907210. doi: 10.1083/jcb.201907210 31727777 PMC7039211

[17] Nielsen J Brandt J Boesen T Hummelshoj T Slaaby R Schluckebier G. Structural Investigations of Full-Length Insulin Receptor Dynamics and Signalling. J Mol Biol (2022) 434:167458. doi: 10.1016/j.jmb.2022.167458 35074483

[18] Lawrence MC. Understanding Insulin and its Receptor From Their Three-Dimensional Structure. Mol Metab (2021) 52:101255. doi: 10.1016/j.molmet.2021.101255 33992784 PMC8513149

[19] Taniguchi CM Emanuelli B Kahn CR. Critical Nodes in Signalling Pathways: Insights Into Insulin Action. Nat Rev Mol Cell Biol (2006) 7:85–96. doi: 10.1038/nrm1837 16493415

[20] Hansen BF Kurtzhals P Jensen AB Dejgaard A Russell-Jones D. Insulin X10 Revisited: A Super-Mitogenic Insulin Analogue. Diabetologia (2011) 54:2226–31. doi: 10.1007/s00125-011-2203-8 21633908

[21] Kurtzhals P Schaffer L Sorensen A Kristensen C Jonassen I Schmid C. Correlations of Receptor Binding and Metabolic and Mitogenic Potencies of Insulin Analogs Designed for Clinical Use. Diabetes (2000) 49:999–1005. doi: 10.2337/diabetes.49.6.999 10866053

[22] Sciacca L Cassarino MF Genua M Pandini G Le Moli R Squatrito S. Insulin Analogues Differently Activate Insulin Receptor Isoforms and Post-Receptor Signalling. Diabetologia (2010) 53:1743–53. doi: 10.1007/s00125-010-1760-6 20424816

[23] Sommerfeld MR Muller G Tschank G Seipke G Habermann P Kurrle R. *In Vitro* Metabolic and Mitogenic Signaling of Insulin Glargine and its Metabolites. PLos One (2010) 5:e9540. doi: 10.1371/journal.pone.0009540 20209060 PMC2832019

[24] Jensen M Hansen B De Meyts P Schaffer L Urso B. Activation of the Insulin Receptor by Insulin and a Synthetic Peptide Leads to Divergent Metabolic and Mitogenic Signaling and Responses. J Biol Chem (2007) 282:35179–86. doi: 10.1074/jbc.M704599200 17925406

[25] Rajapaksha H Forbes BE. Ligand-Binding Affinity at the Insulin Receptor Isoform-A and Subsequent IR-A Tyrosine Phosphorylation Kinetics are Important Determinants of Mitogenic Biological Outcomes. Front Endocrinol (2015) 6:107. doi: 10.3389/fendo.2015.00107 PMC4493403 26217307

[26] Jensen M De Meyts P. Molecular Mechanisms of Differential Intracellular Signaling From the Insulin Receptor. Vitamin Hormone (2009) 80:51–75. doi: 10.1016/S0083-6729(08)00603-1 19251034

[27] Bedinger DH Goldfine ID Corbin JA Roell MK Adams SH. Differential Pathway Coupling of the Activated Insulin Receptor Drives Signaling Selectivity by XMetA, an Allosteric Partial Agonist Antibody. J Pharmacol Exp Ther (2015) 353:35–43. doi: 10.1124/jpet.114.221309 25613982

[28] Yunn NO Koh A Han S Lim JH Park S Lee J. Agonistic Aptamer to the Insulin Receptor Leads to Biased Signaling and Functional Selectivity Through Allosteric Modulation. Nucleic Acids Res (2015) 43:7688–701. doi: 10.1093/nar/gkv767 PMC4652772 26245346

[29] Pillutla RC Hsiao KC Beasley JR Brandt J Ostergaard S Hansen PH. Peptides Identify the Critical Hotspots Involved in the Biological Activation of the Insulin Receptor. J Biol Chem (2002) 277:22590–4. doi: 10.1074/jbc.M202119200 11964401

[30] Ong SC Belgi A van Lierop B Delaine C Andrikopoulos S MacRaild CA. Probing the Correlation Between Insulin Activity and Structural Stability Through Introduction of the Rigid A6-A11 Bond. J Biol Chem (2018) 293:11928–43. doi: 10.1074/jbc.RA118.002486 PMC6066309 29899115

[31] Van Lierop BJ Bornschein C Jackson WR Robinson AJ. Ring-Closing Metathesis in Peptides – the Sting is in the Tail! Aust J Chem (2011) 64:806–11. doi: 10.1071/CH11090

[32] Hansen BF Glendorf T Hegelund AC Lundby A Lutzen A Slaaby R. Molecular Characterisation of Long-Acting Insulin Analogues in Comparison With Human Insulin, IGF-1 and Insulin X10. PLos One (2012) 7:e34274. doi: 10.1371/journal.pone.0034274 22590494 PMC3348127

[33] Denley A Bonython ER Booker GW Cosgrove LJ Forbes BE Ward CW. Structural Determinants for High-Affinity Binding of Insulin-Like Growth Factor II to Insulin Receptor (IR)-A, the Exon 11 Minus Isoform of the IR. Mol Endocrinol (2004) 18:2502–12. doi: 10.1210/me.2004-0183me.2004-0183 15205474

[34] Cross DA Alessi DR Cohen P Andjelkovich M Hemmings BA. Inhibition of Glycogen Synthase Kinase-3 by Insulin Mediated by Protein Kinase B. Nature (1995) 378:785–9. doi: 10.1038/378785a0 8524413

[35] Hamer I Foti M Emkey R Cordier-Bussat M Philippe J De Meyts P. An Arginine to Cysteine(252) Mutation in Insulin Receptors From a Patient With Severe Insulin Resistance Inhibits Receptor Internalisation But Preserves Signalling Events. Diabetologia (2002) 45:657–67. doi: 10.1007/s00125-002-0798-5 12107746

[36] Ceresa BP Kao AW Santeler SR Pessin JE. Inhibition of Clathrin-Mediated Endocytosis Selectively Attenuates Specific Insulin Receptor Signal Transduction Pathways. Mol Cell Biol (1998) 18:3862–70. doi: 10.1128/MCB.18.7.3862 PMC108970 9632770

[37] Daunt DA Hurt C Hein L Kallio J Feng F Kobilka BK. Subtype-Specific Intracellular Trafficking of Alpha2-Adrenergic Receptors. Mol Pharmacol (1997) 51:711–20. doi: 10.1124/mol.51.5.711 9145909

[38] Rakatzi I Ramrath S Ledwig D Dransfeld O Bartels T Seipke G. A Novel Insulin Analog With Unique Properties: LysB3,GluB29 Insulin Induces Prominent Activation of Insulin Receptor Substrate 2, But Marginal Phosphorylation of Insulin Receptor Substrate 1. Diabetes (2003) 52:2227–38. doi: 10.2337/diabetes.52.9.2227 12941761

[39] Choi E Kikuchi S Gao H Brodzik K Nassour I Yopp A. Mitotic Regulators and the SHP2-MAPK Pathway Promote IR Endocytosis and Feedback Regulation of Insulin Signaling. Nat Commun (2019) 10:1473. doi: 10.1038/s41467-019-09318-3 30931927 PMC6443781

[40] Hall C Yu H Choi E. Insulin Receptor Endocytosis in the Pathophysiology of Insulin Resistance. Exp Mol Med (2020) 52:911–20. doi: 10.1038/s12276-020-0456-3 PMC7338473 32576931

[41] Zinkle A Mohammadi M. A Threshold Model for Receptor Tyrosine Kinase Signaling Specificity and Cell Fate Determination. F1000Res (2018) 7:F1000 Faculty Rev-872. doi: 10.12688/f1000research.14143.1 PMC6013765 29983915

[42] Soos MA Siddle K Baron MD Heward JM Luzio JP Bellatin J. Monoclonal Antibodies Reacting With Multiple Epitopes on the Human Insulin Receptor. Biochem J (1986) 235:199–208. doi: 10.1042/bj2350199 2427071 PMC1146668

[43] Soos MA Siddle K. Immunological Relationships Between Receptors for Insulin and Insulin-Like Growth Factor I. Evidence for Structural Heterogeneity of Insulin-Like Growth Factor I Receptors Involving Hybrids With Insulin Receptors. Biochem J (1989) 263:553–63. doi: 10.1042/bj2630553 PMC1133463 2480779

[44] Soos MA Field CE Lammers R Ullrich A Zhang B Roth RA. A Panel of Monoclonal Antibodies for the Type I Insulin-Like Growth Factor Receptor. Epitope Mapping, Effects on Ligand Binding, and Biological Activity. J Biol Chem (1992) 267:12955–63. doi: 10.1016/S0021-9258(18)42367-8 1377676

[45] Ganderton RH Stanley KK Field CE Coghlan MP Soos MA Siddle K. A Monoclonal Anti-Peptide Antibody Reacting With the Insulin Receptor Beta-Subunit. Characterization of the Antibody and its Epitope and Use in Immunoaffinity Purification of Intact Receptors. Biochem J (1992) 288(Pt 1):195–205. doi: 10.1042/bj2880195 1280110 PMC1132099

[46] Sell C Dumenil G Deveaud C Miura M Coppola D DeAngelis T. Effect of a Null Mutation of the Insulin-Like Growth Factor I Receptor Gene on Growth and Transformation of Mouse Embryo Fibroblasts. Mol Cell Biol (1994) 14:3604–12. doi: 10.1128/mcb.14.6.3604-3612.1994 PMC358728 8196606

[47] Pietrzkowski Z Lammers R Carpenter G Soderquist AM Limardo M Phillips PD. Constitutive Expression of Insulin-Like Growth Factor 1 and Insulin-Like Growth Factor 1 Receptor Abrogates All Requirements for Exogenous Growth Factors. Cell Growth Differ (1992) 3:199–205. 1325181

[48] Gauguin L Delaine C Alvino CL McNeil KA Wallace JC Forbes BE. Alanine Scanning of a Putative Receptor Binding Surface of Insulin-Like Growth Factor-I. J Biol Chem (2008) 283:20821–9. doi: 10.1074/jbc.M802620200 PMC3258947 18502759

[49] Gauguin L Klaproth B Sajid W Andersen AS McNeil KA Forbes BE. Structural Basis for the Lower Affinity of the Insulin-Like Growth Factors for the Insulin Receptor. J Biol Chem (2008) 283:2604–13. doi: 10.1074/jbc.M709220200 18048361

[50] Hvid H Glendorf T Brandt J Slaaby R Lutzen A Kristensen K. Increased Insulin Receptor Binding and Increased IGF-1 Receptor Binding are Linked With Increased Growth of L6hIR Cell Xenografts *In Vivo* . Sci Rep (2020) 10:7247. doi: 10.1038/s41598-020-64318-4 32350367 PMC7190841

[51] Govers R Coster AC James DE. Insulin Increases Cell Surface GLUT4 Levels by Dose Dependently Discharging GLUT4 Into a Cell Surface Recycling Pathway. Mol Cell Biol (2004) 24:6456–66. doi: 10.1128/MCB.24.14.6456-6466.2004 PMC434240 15226445

[52] Modan-Moses D Janicot M McLenithan JC Lane MD Casella SJ. Expression and Function of Insulin/Insulin-Like Growth Factor I Hybrid Receptors During Differentiation of 3T3-L1 Preadipocytes. Biochem J (1998) 333(Pt 3):825–31. doi: 10.1042/bj3330825 PMC1219650 9677346

[53] van Dam EM Govers R James DE. Akt Activation is Required at a Late Stage of Insulin-Induced GLUT4 Translocation to the Plasma Membrane. Mol Endocrinol (2005) 19:1067–77. doi: 10.1210/me.2004-0413 15650020

