# Supplementary material for: Minimizing Mitogenic Potency of Insulin Analogues Through Modification of a Disulfide Bond
Source: Front Endocrinol (Lausanne). 2022 Jun 27;13:907864. doi: 10.3389/fendo.2022.907864 (PMC9271792; doi:10.3389/fendo.2022.907864)
Supplement: Supplementary file 1 [file DataSheet_1.pdf]

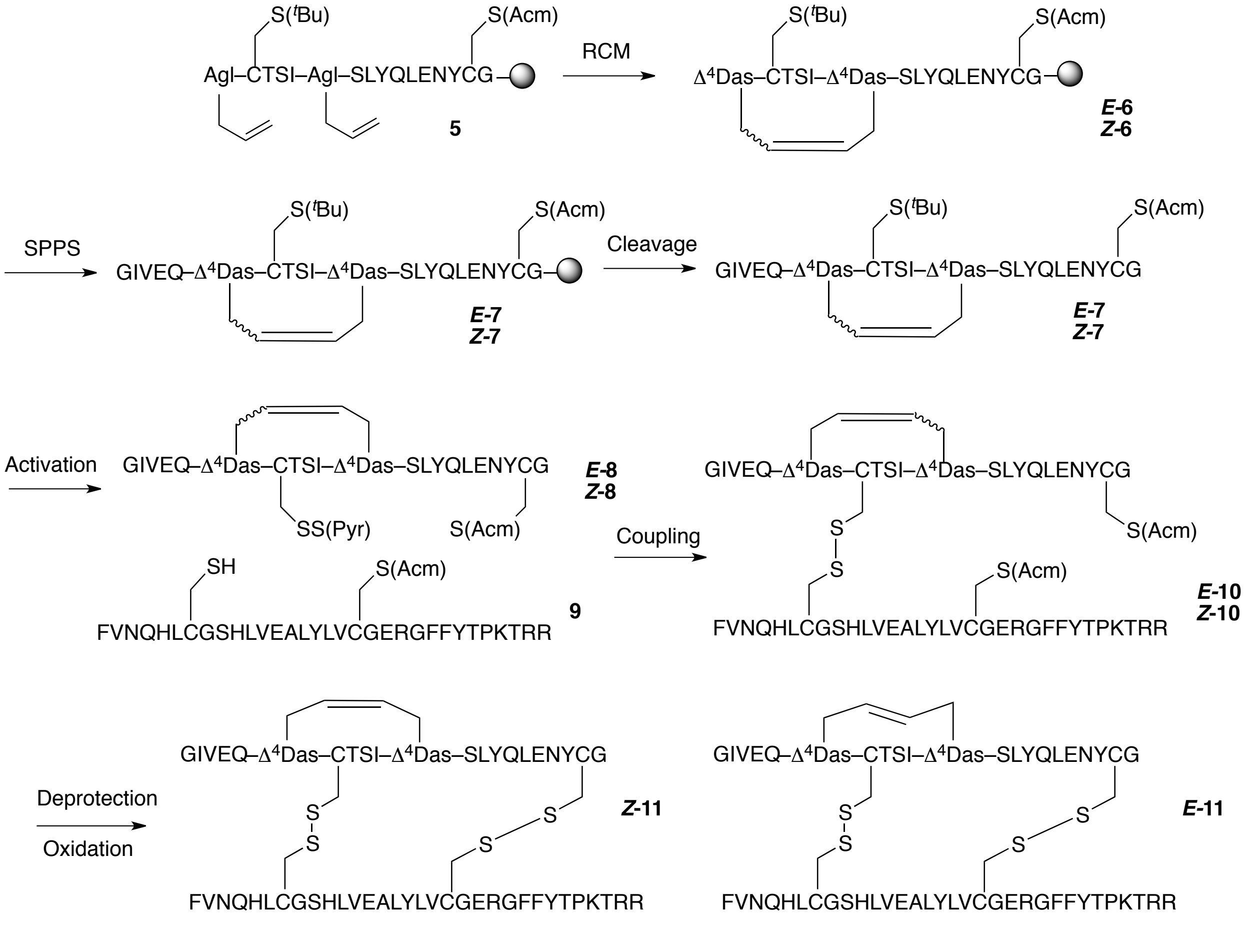

Figure S1. Synthesis of *c*[Δ4A6,11]-Dicarba glargine insulin (*E*-11).

Synthesis of dicarba glargine insulins was performed *via* ring-closing metathesis (RCM) and an interrupted solid phase peptide synthesis (SPPS)-catalysis approach. L-Allylglycine (Agl), tert-butyl (^{t}Bu), acetamidomethyl (Acm), pyridinyl (Pyr), *cis* isomer (*Z*), *trans* isomer (*E*).

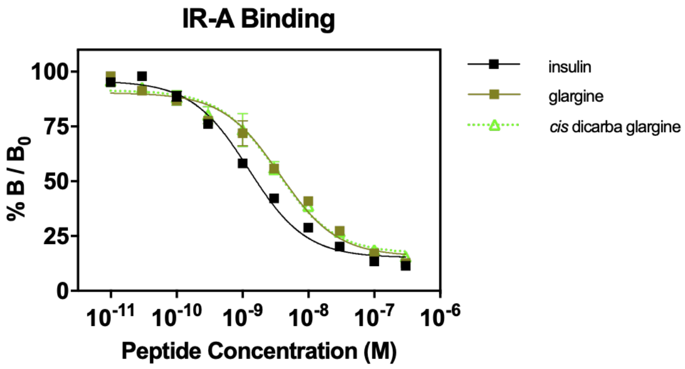

	IC_{50} (nM)	Affinity (% insulin ± SEM)
Insulin	1.29±0.05	100
Glargine	3.67±0.15	33
cis dicarba glargine	3.31±0.29	37

Figure S2. Receptor binding affinity of insulin, glargine and *cis* dicarba glargine analogues on the IR-A.

Competition binding of insulin, glargine and *cis* dicarba glargine analogues with europium-labelled insulin for the IR-A. Results are expressed as a percentage of binding in the absence of competing ligand (%B/B_0). IR-A binding: insulin *vs* glargine **** ($P = 0.0001$); insulin *vs cis* dicarba glargine **** ($P \leq 0.0001$); glargine *vs cis* dicarba glargine (ns). (2-way ANOVA; Tukey's multiple comparison). All data are the mean ± S.E.M. n = 5 or 6 for each data point, with data derived from 2 independent experiments. Error bars are shown when greater than the size of the symbols.

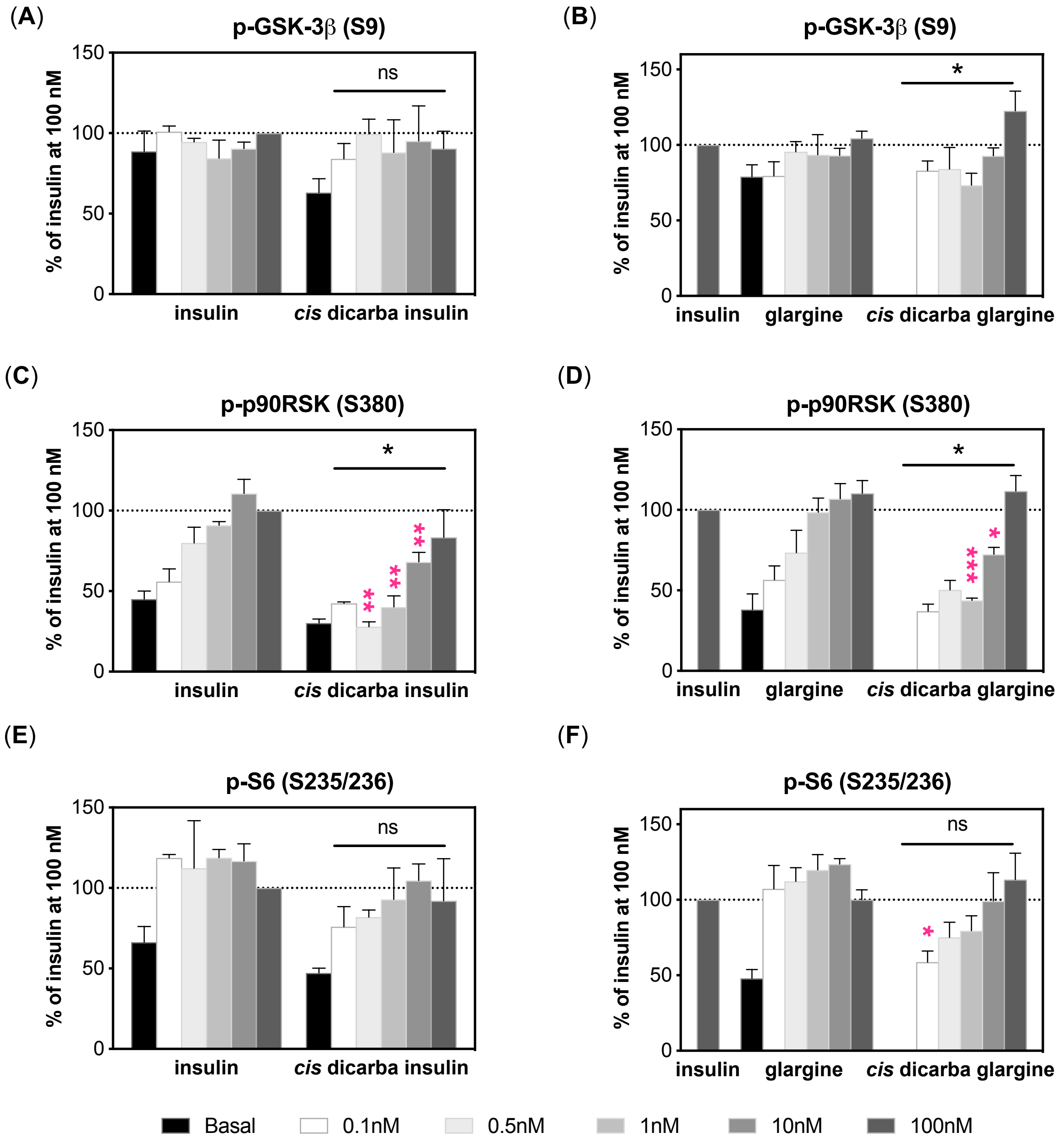

Figure S3. Quantitation of specific phosphorylation on GSK, p90RSK and S6 proteins in IR-A overexpressing L6 myoblasts stimulated by insulin, glargine and *cis* dicarba analogues in increasing concentrations.

Quantitation of western blots presented in *Fig. 4A-D,* representative blots of n = at least 3 independent experiments. Serum starved IR-A overexpressing L6 myoblasts were stimulated with increasing concentrations (0, 0.1, 0.5, 1, 10 and 100 nM) of insulin, *cis* dicarba insulin, glargine or *cis* dicarba glargine for 10 min. Phosphorylation levels are expressed as percentage of level detected when cells were stimulated with 100 nM of insulin for 10 min; normalized to β-tubulin: (*A-B*) GSK-3β (phospho-Ser9), (*C-D*) p90RSK (phospho-Ser380) and (*E-F*) S6 ribosomal (phospho-Ser235/236). All data are the mean ± S.E.M presented as bar graphs. Statistical significance of the overall difference in phosphorylation levels stimulated by *cis* dicarba insulin compared to native insulin (or *cis* dicarba glargine compared to glargine insulin) were determined *via* 2-way ANOVA (bars above graph); difference comparing each stimulating concentration (pink asterisks) were further analyzed using Holm-Sidak test. ns: non significant; * ($P \leq 0.05$), ** ($P \leq 0.01$); *** ($P \leq 0.001$); **** ($P \leq 0.0001$).

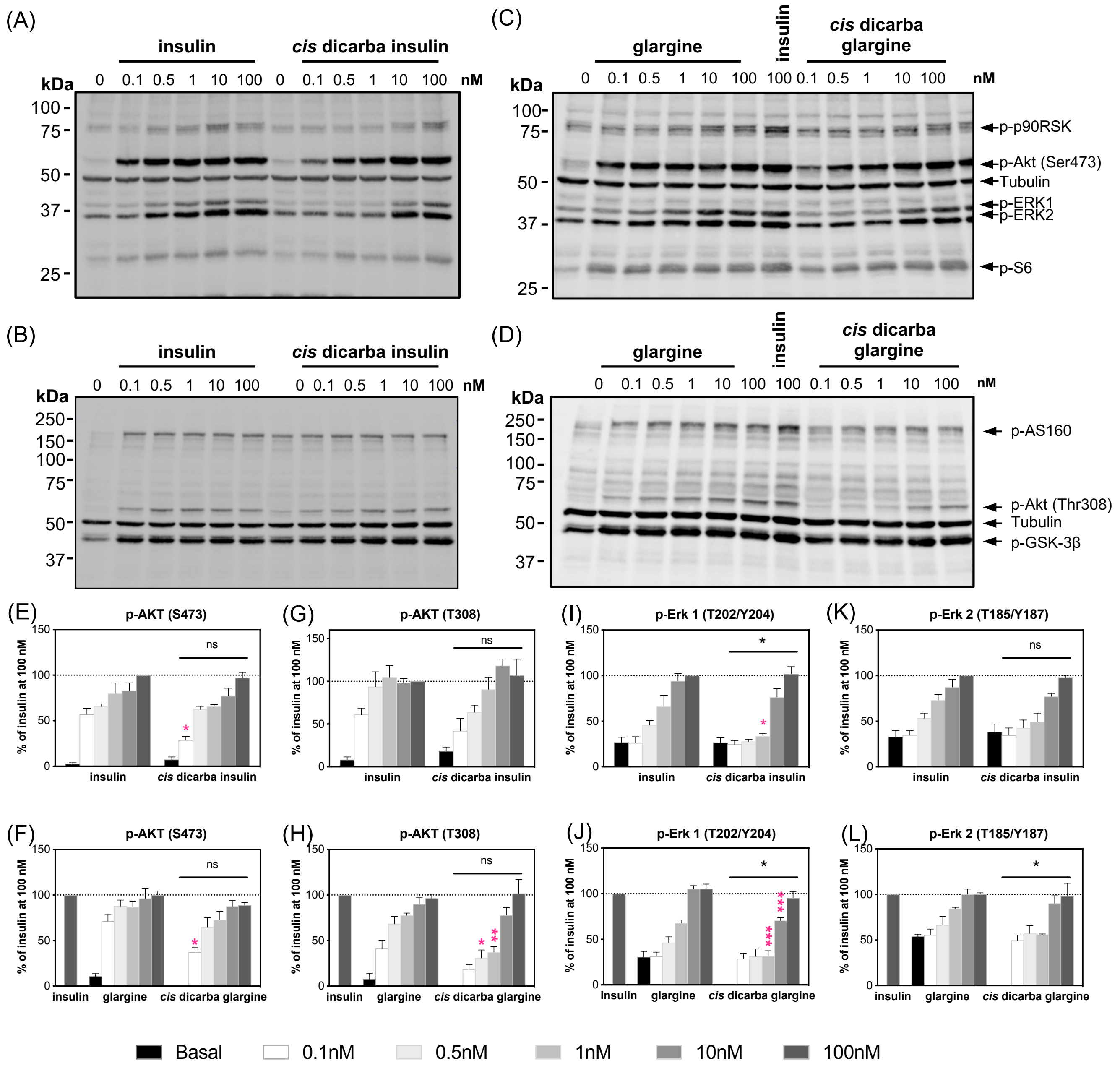

Figure S4. ***Cis*** **dicarba insulin and** ***cis*** **dicarba glargine insulin exhibit dose-dependent signaling bias** ***via*** **IR-B overexpressing fibroblasts.**

Serum starved R⁻IR-B fibroblasts were stimulated with increasing concentrations (0, 0.1, 0.5 1, 10 and 100 nM) of (*A-B*) insulin, *cis* dicarba insulin, (*C-D*) insulin glargine or *cis* dicarba glargine for 10 min. (*E-L*): Quantitation of western blots presented in (*A-D*, representative blots of n = at least 3 independent experiments). Phosphorylation levels are expressed as percentage of level detected when cells were stimulated with 100 nM of insulin for 10 min; normalized to β-tubulin: (*E-F*) Akt (phosho-Ser473), (*G-H*) Akt (phosho-Thr308), (*I-J*) ERK 1 (phospho-Thr202/Tyr204) and (*K-L*) ERK2 (phsopho-Thr185/Tyr187). Quantitation of other phosphorylated proteins are included in Fig. S5. All data are the mean ± S.E.M. Statistical significance of the overall difference in phosphorylation levels stimulated by *cis* dicarba insulin compared to native insulin or *cis* dicarba glargine compared to glargine insulin were determined *via* 2-way ANOVA (bars above graph); difference comparing each stimulating concentration (pink asterisks) were further analyzed using Holm-Sidak test. ns: non-significant; * ($P \leq 0.05$), ** ($P \leq 0.01$); *** ($P \leq 0.001$); **** ($P \leq 0.0001$).

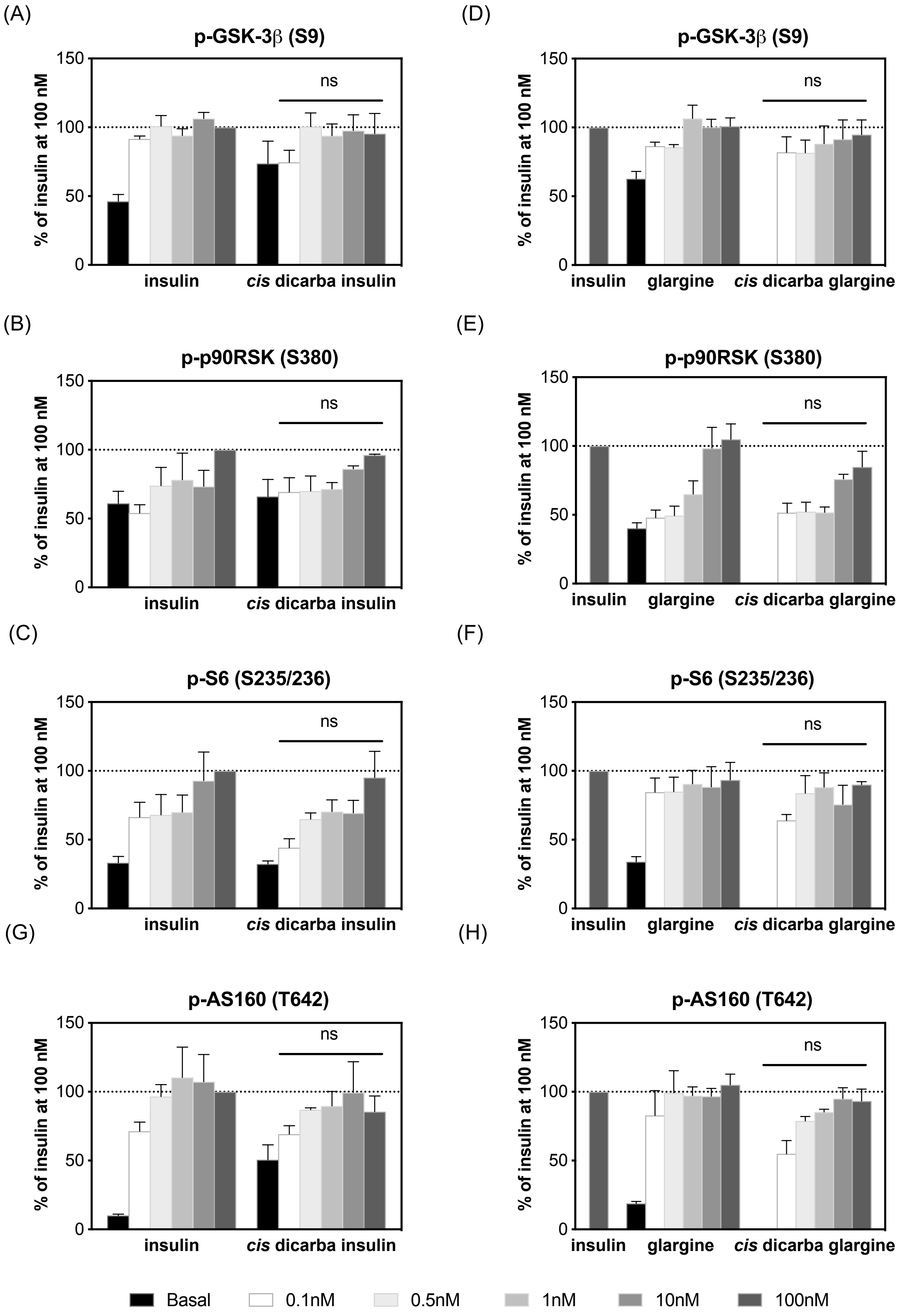

Figure S5. Quantitation of specific phosphorylation on GSK, p90RSK, S6 and AS160 proteins in IR-B overexpressing fibroblasts stimulated by insulin, glargine and *cis* dicarba analogues in increasing concentrations.

Quantitation of western blots presented in Fig. S4. Serum starved R^{-}IR-B fibroblasts were stimulated with increasing concentrations (0, 0.1, 0.5, 1, 10 and 100 nM) of insulin, *cis* dicarba insulin, glargine or *cis* dicarba glargine for 10 min. Phosphorylation levels of (*A-B*) GSK-3β (phospho-Ser9), (*C-D*) p90RSK (phospho-Ser380) (*E-F)* S6 ribosomal (phospho-Ser235/236) and (*G-H*) pAS160 (phospho-Thr642) are expressed as percentage of level detected when cells were stimulated with 100 nM of insulin for 10 min. All data are the mean ± S.E.M presented as bar graphs. n = at least 3 independent experiments. In each case, data are normalized to the loading control β-tubulin. Statistical significance of the overall difference in phosphorylation levels stimulated by *cis* dicarba insulin compared to native insulin (or *cis* dicarba glargine compared to glargine insulin) were determined *via* 2-way ANOVA (bars above graph); difference comparing each stimulating concentration (pink asterisks) were further analyzed using Holm-Sidak test. ns: non significant; * ($P \leq 0.05$), ** ($P \leq 0.01$); *** ($P \leq 0.001$); **** ($P \leq 0.0001$).

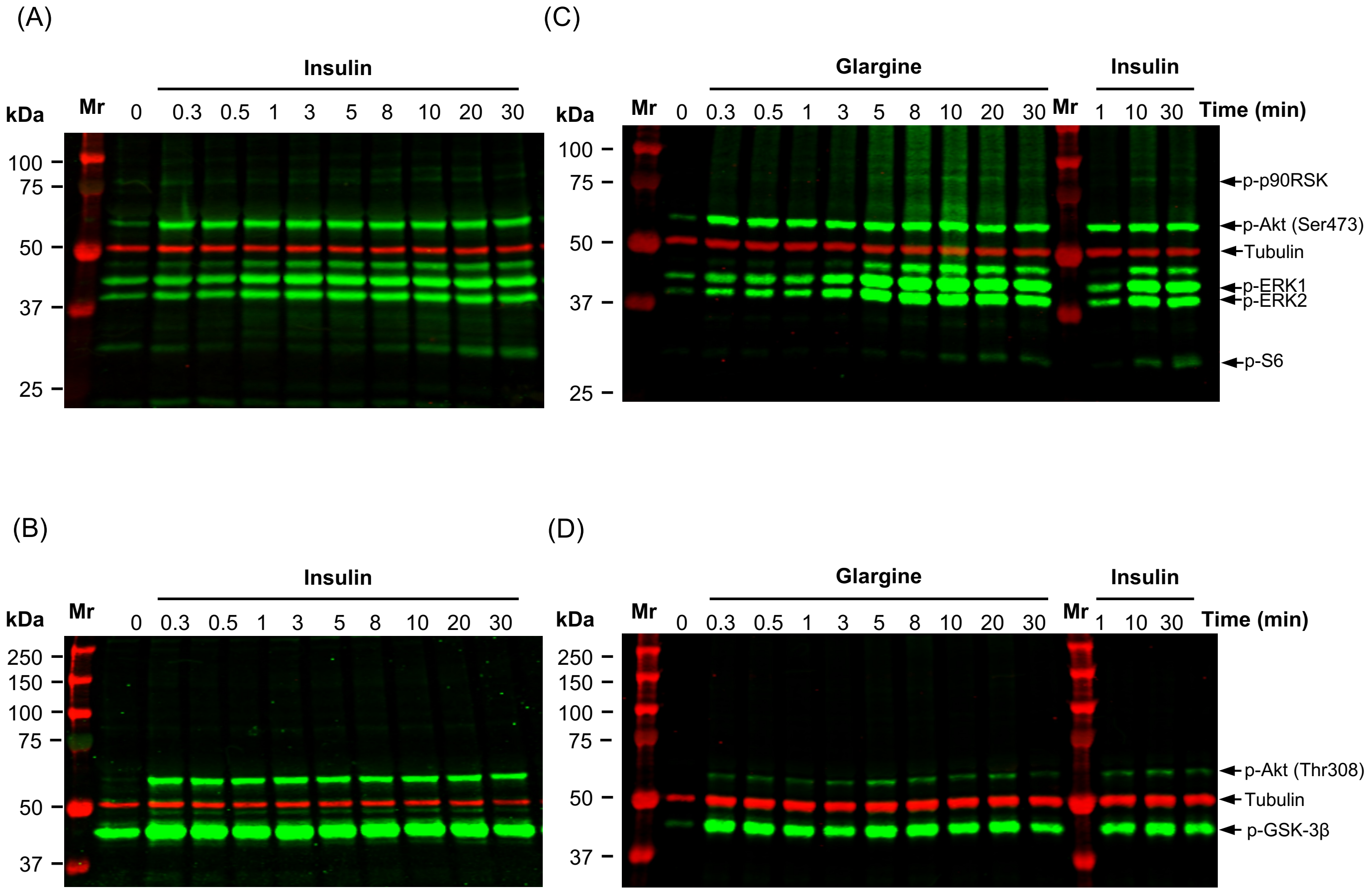

Figure S6. Representative western blot of IR-A stimulated by 10 nM of insulin or glargine insulin in time-course analyses.

Serum starved IR-A overexpressing L6 myoblasts (hIR-A L6) were stimulated with 10 nM of (*A-B*) insulin or (*C-D*) glargine insulin in a time-course of t = 0, 0.33 (20 s), 0.5 (30 s), 1, 3, 5, 8, 10, 20 and 30 min. Quantitation of western blots can be seen in Fig. S7.

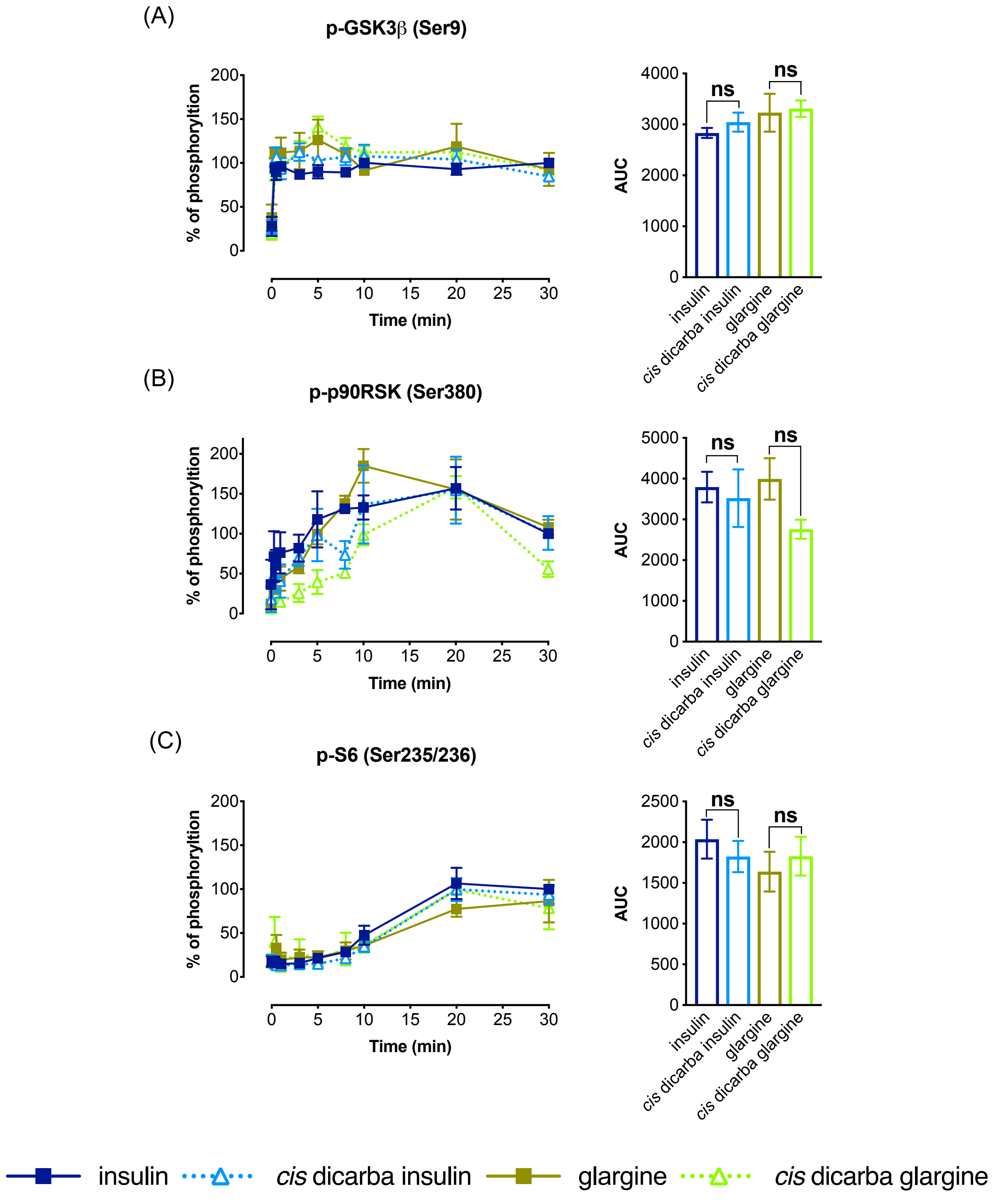

Figure S7. Quantitation of specific phosphorylation on GSK, p90RSK and S6 proteins in IR-A overexpressing L6 myoblasts stimulated by insulin, glargine and *cis* dicarba analogues in time-dependent analyses.

Quantitation of western blots presented in Fig. 5 and S6. Serum starved IR-A overexpressing L6 myoblasts (hIR-A L6) were stimulated with 10 nM of insulin (Fig. S6, *A-B*), glargine (Fig. S6, *C-D*), *cis* dicarba insulin (Fig. 5, *A-B*) or *cis* dicarba glargine (Fig. 5, *C-D*) in a time-course of $t = 0$, 0.33 (20 s), 0.5 (30 s), 1, 3, 5, 8, 10, 20 and 30 min. Phosphorylation levels are expressed as percentage of level detected when cells were stimulated with 10 nM of insulin for 30 min; normalized to β-tubulin : (*A*) GSK-3β (phospho-Ser9), (*B*) p90RSK (phospho-Ser380) and (*C*) S6 ribosomal (phospho-Ser235/236). All data are the mean ± S.E.M presented as XY graphs. Error bars are shown when greater than the size of the symbols. AUC, area under the curves derived from % of phosphorylation over 30 minutes. Statistical significance of the difference in total phosphorylation over 30 minutes measured as area under curves when stimulated by *cis* dicarba insulin compared to insulin or *cis* dicarba glargine compared to glargine insulin were determined *via* ordinary one-way ANOVA. ns: non-significant; * ($P \leq 0.05$), ** ($P \leq 0.01$); *** ($P \leq 0.001$); **** ($P \leq 0.0001$).
